# Dysregulated glycerophospholipid metabolism in amygdala may mediate favipiravir-induced anxiety-like behaviors in mice

**DOI:** 10.3389/fphar.2025.1491150

**Published:** 2025-03-04

**Authors:** Yuzhou Xiao, Chunqi Liu, Xiaojie Wang, Hongchun Li, Liang Wang, Kun Gou, Xingchen Liu, Xinqi Guan, Xia Zhou, Xiumei He, Yue Zhao, Lei Tao, Xiaodan Pan, Linhong Jiang, Yaxing Chen, Huan Liu, Yanping Dai, Qian Bu, Meng Qin, Ruiming Zhu, Bo Chen, Angelo D. Flores, Yinglan Zhao, Xiaobo Cen

**Affiliations:** ^1^ Mental Health Center and National Chengdu Center for Safety Evaluation of Drugs, State Key Laboratory of Biotherapy/Collaborative Innovation Center for Biotherapy, West China Hospital, Sichuan University, Chengdu, China; ^2^ West China School of Pharmacy, Sichuan University, Chengdu, China; ^3^ School of Life Sciences, Guangxi Normal University, Guilin, China; ^4^ Chengdu Westchina Frontier Pharmatech, Co., Ltd., Chengdu, China; ^5^ Department of Neuroscience, City University of Hong Kong, Kowloon, China

**Keywords:** favipiravir, anxiety-like behaviors, amygdala, glycerophospholipid metabolism, neurophysiological

## Abstract

Favipiravir, the first RNA polymerase inhibitor approved to treat resistant influenza, has been reported to be associated with central nervous system (CNS) side effects, particularly anxiety-like behavior; nevertheless, the underlying mechanism remains largely unknown. In this study, we investigated the effect of favipiravir on the neurobehavior of mice, and combined lipidomics and transcriptomics analysis to explore the mechanism underlying this effect. In behavioral tests, the mice displayed anxiety-like behaviors after oral favipiravir administration (200 mg/kg) for 7 days continuously. By lipidomics analysis, we observed that favipiravir induced a dysregulation of glycerophospholipid metabolism in the amygdala. Moreover, favipiravir significantly reduced the mRNA level of glycerol-3-phosphate acyltransferase 2 (*Gpat2*), the rate-limiting enzyme of glycerophospholipid synthesis. Notably, favipiravir markedly reduced the levels of docosahexaenoic acid-enriched phosphatidylethanolamine or phosphatidylcholine (DHA-PE/PC) and arachidonic acid-enriched phosphatidylethanolamine or phosphatidylcholine (AA-PE/PC), two components of glycerophospholipids, in the amygdala. The increased expression of phospholipase A2 (*Pla2*) may attribute to the enhanced release of arachidonic acid (AA) from AA-PE/PC. Furthermore, favipiravir altered neurite morphology and reduced neurophysiological activity in amygdala neurons *in vitro*. Collectively, dysregulated glycerophospholipid metabolism in the amygdala may contribute to the adverse effect of favipiravir.

## Highlights

- Favipiravir induces anxiety-like behavior in mice following 7 days of treatment.
- Favipiravir alters GP metabolism in the amygdala, reducing unsaturation and levels of DHA-PE/PC and AA-PE/PC.
- Favipiravir decreases the mRNA expression of GPAT2, a key enzyme in GP synthesis, while increasing the expression of PLA2 isoforms involved in AA release.
- Favipiravir impacts axonal mitochondrial structure, and synaptic vesicles, potentially impairing neurophysiological activity.

## 1 Introduction

Favipiravir (FVP) (Shrestha et al., 2020), an RNA polymerase inhibitor, inhibits virus replication by targeting the RNA-dependent RNA polymerase (RdRp) enzyme in human cells (Aktaş et al., 2021). It was first developed in 2014 to treat influenza strains that were resistant to neuraminidase inhibitors (Furuta et al., 2017). Favipiravir has been used for the treatment of life-threatening infections of fatal infections caused by Ebola, rabies, norovirus and SARS-CoV-2 (De Clercq, 2019). Although favipiravir is considered a relatively safe drug, there have been reports of side effects associated with the use of favipiravir, like elevated liver enzymes, nausea and vomiting, tachycardia, and diarrhea (Kaur et al., 2020).

Due to the pandemic of COVID-19, clinical studies on favipiravir have increased, and it has also shown potential neurological side effects. Meta-analysis of clinical trials on the safety of favipiravir revealed that approximately 7%–12% of patients experienced different degrees of neurological adverse effects (Shah et al., 2022; Solaymani-Dodaran et al., 2021). At the same time, Chen et al. (2020) found that favipiravir treatment caused psychiatric symptoms in 5 patients (5/116), while only one case (1/120) occurred in the Arbidol treatment group (Chen et al., 2021). Neurological side effects caused by favipiravir, including agitation, recurrent paranoid delusions, sleep disturbances, auditory hallucinations and anxiety (Hassanipour et al., 2021; Madelain et al., 2020a; Ueda et al., 2022; Duyan and Ozturan, 2021). However, the pathophysiological mechanism of the neurological side effects associated with the use of favipiravir is not fully known and was considered to be worthy of investigation.

Structurally, favipiravir is a pyrazinamide derivative that can cross the blood-brain barrier (Madelain et al., 2020b). A recent study showed that pyrazinamide causes neurotoxicity in juvenile zebrafish, which is evidenced by the markedly decreased motor capacity and length of the dopaminergic neuron-rich brain region (Wang et al., 2022). Moreover, antiviral medications may affect the production, transport and metabolism of lipids, causing dysfunction in lipid homeostasis. For instance, Oseltamivir, an RNA polymerase inhibitor, sialylates serum glycolipids to activate brain D2 receptors, which may be linked to neuropsychiatric adverse events (Suzuki and Masuda, 2008). Also, antiretroviral dramatically increases the concentration of 4-hydroxynonenal, a peroxidation product of arachidonic acid, in the hippocampus of mice, suggesting disturbed lipid metabolism in the brain (Zulu et al., 2021). Therefore, we hypothesize that favipiravir-related CNS side effects may be related to changes in lipid homeostasis.

The brain is the second most lipid-rich organ after adipose tissue and is diverse in its composition (Raulin et al., 2022). Lipids play a variety of roles in the brain, including membrane dynamics, neurotransmission, and synapse formation (Garcia Corrales et al., 2021). Clinical investigations have employed lipidomics to detect and diagnose CNS diseases, adding a new dimension to the research on CNS diseases (Wang et al., 2018; Liu et al., 2021). It is known that neuronal dysfunction and cognitive decline are associated with the disruption of brain lipid homeostasis in certain brain regions (Shinohara et al., 2017; Sienski et al., 2021; Kao et al., 2020; Steen et al., 2017). It is unclear whether favipiravir causes abnormal lipid homeostasis in specific brain regions.

Based on the changes in behavioral preferences in experimental animal models, our results show that favipiravir causes anxiety-like behaviors in mice. The amygdala is a key brain region responsible for processing and regulating emotions, including fear and anxiety (Chiurchiù and Maccarrone, 2016). The lipid metabolism in the amygdala plays an important role in regulating mood and anxiety (Hu et al., 2022). Disruption of lipid metabolism in the amygdala impairs neuronal excitability, synaptic plasticity, and processing of emotional information (Gunduz-Cinar et al., 2013; Coker et al., 2021). By combining unbiased lipidomic and mRNA-sequence analyses, we investigated how favipiravir affects amygdala lipid homeostasis. Our findings reveal that favipiravir causes anxiety-like behavior in mice, which may be associated with dysregulated glycerophospholipid metabolism in the amygdala.

## 2 Materials and methods

### 2.1 Drugs

Favipiravir (S7975, Selleck) was suspended in pure water containing 0.5% sodium carboxymethyl cellulose. The PLA2 inhibitor Bromoenol lactone (HY-107411, BEL) was obtained from MedChemExpress and dissolved in DMSO.

### 2.2 Preparation of liposomes

Liposomes were prepared following the previously described method with slight modifications (Hossain et al., 2006). Initially, a mixture of DHA-PE (850797C, Sigma) (2 mg) and cholesterol (molar ratio 1:1) was completely dissolved in 3 mL of chloroform and then transferred to a 25 mL round-bottom flask. Chloroform was completely removed using a rotary evaporator in a 25°C water bath, forming a lipid film in the flask. The flask containing the lipid film was placed in a vacuum drying oven at room temperature to dry overnight. After adding phosphate buffer, the flask was stirred with a magnetic stirrer for 30 min, followed by ultrasonication for 10 min in an ice bath. A liposome extruder was used to pass the liposome solution through a 100 nm pore size membrane 20 times. Prior to use, the liposomes were filtered through a 0.22 μm pore size membrane. DHA-PL liposomes were freshly prepared each time, and their particle size was measured before each experiment (Figure 7E).

### 2.3 Animals and drug administration

Adult male C57BL/6 mice (6–8 weeks old, 18–22 g) were obtained from Vital River Laboratory Animal Technology Co., Ltd. (Beijing, China). The mice were housed under standard conditions (12-h light/dark cycle, lights on from 07:00 to 19:00) at a constant room temperature, with food and water available *ad libitum*. All procedures were approved by the Sichuan University Institutional Animal Care and Use Committee and accredited by the Association for Assessment and Accreditation of Laboratory Animal Care (AAALAC). The mice were randomly divided into four groups, with ten mice in each group. One group received the solvent, while the other three groups received favipiravir treatment. Mice received intragastric (i.g.) administration of favipiravir (200 mg/kg) for 3, 5, and 7 consecutive days, respectively. The administration method and dosage of favipiravir followed clinical guidelines, with the dosage for mice calculated based on the equivalent dose ratio derived from the body surface area conversion between humans and mice (Tani et al., 2018; Zhu et al., 2018).

### 2.4 Neurobehavioral tests

The dark/light box test was used to assess the anxiety behaviors of the mice. There were two identical boxes (25 × 25 cm), one light box with direct lighting of 170 LUX intensity and one dark box with black acrylic. There is a channel between the two boxes through which mice can move freely. After the mice were placed in the dark box, the time spent in the light box and the number of transitions between the two chambers were recorded by the camera within 5 min. The open field test was also used to examine the anxiety-like behaviors of the mice. The 48 × 48 × 30 cm black acrylic box was divided into the center and the periphery using a 16-beam animal activity monitor. EthoVision software (Noldus Information Technology) was used to analyze the behaviors of each mouse.

The forced swimming test was used to assess depression and despair in mice. Mice were placed in experimental room for 1 h for habituation. During the experiment, the mice were placed in a transparent cylindrical container with an opening of 15 cm in diameter and a water depth of 15 cm. The water temperature was set at 23°C ± 2 °C. The whole process was recorded by the camera, and the total time of immobility of the mouse within 5 min was calculated.

### 2.5 Blood biochemistry and histopathology examination

Two hours after completion of the behavioral experiment, the mice were immediately euthanized by rapid decapitation, and blood was taken to measure serum biochemistry. The brain and other major organs were dissected for lipidomic analysis, mRNA-seq analysis, and histopathological examination. An automatic biochemical analyzer (Cobas) was used to measure serum biochemistry, including total protein (TP), albumin (ALB), globulin (GLB), alkaline phosphatase (ALP), aspartate aminotransferase (AST), alanine aminotransferase (ALT), glucose (GLU), urea (UREA), cholesterol (CHOL), triglyceride (TG), and creatinine (CREA). The fixed tissue was embedded in paraffin and cut into 5 µm thick slices, followed by hematoxylin and eosin staining.

### 2.6 Lipidomics analysis

#### 2.6.1 Lipid extraction from the amygdala

Mouse amygdala lipids were extracted using methyl tert-butyl ether (MTBE) lipid extraction method (Yu et al., 2020; Aldana et al., 2020). Briefly, prechilled methanol (150 μL) and MTBE (450 μL) were added sequentially to the frozen tissue (25–30 mg). After incubating the mixture at room temperature for 10 min, amygdala was homogenized with a bead-based homogenizer for three cycles of 15 s and 20 s intervals at 6,500 rpm. The homogeneous mixture was then treated with 300 μL of 25% methanol diluted in sterile Milli-Q water to induce phase separation, followed by centrifugation at 14,000 g for 10 min. The upper organic phase was carefully aspirated without disturbing the middle layer. The extracted oil was evaporated at room temperature with a gentle stream of nitrogen and then kept at 80°C until analysis.

#### 2.6.2 Lipidomics chromatographic conditions

Mobile phase A1 was 4:6 acetonitrile: water with 10 mM ammonium acetate, and mobile phase B1 was 1:9 acetonitrile: isopropyl alcohol with 10 mM ammonium acetate. Chromatography was performed on a Waters Spectra C18 column at a flow of 0.3 mL/min with an injection volume of 3 μL.

#### 2.6.3 Lipidomics mass spectrometry conditions

The mass spectrometry analysis was performed in both positive and negative ESI modes. Relevant instrumental parameters were set as follows: scan time, 36 min; scan range, 50–1,200 kDa; capillary voltage, 2.0 kV; cone voltage: 30 V; sampling cone: 30; source block and desolvation gas temperatures, 150 °C and 400 °C; flow rate of desolvent gas: 700 L/h; cone-hole gas flow rate: 50 L/h; low impact energy: 6 E V; and high impact energy: 25–30 E V.

#### 2.6.4 Data processing and analysis

Lipid Search v4.2.21 analytical software (ThermoFisher Scientific) was used to identify the lipidomics. The absolute intensities of all identified compounds (normalized abundance) were adjusted to the relative abundances of lipid molecules using data sheets from the Lipid Search software (compared to the control group). Pareto scaling was used for the final statistical model. To create group clusters, the data were processed using supervised partial least-squares discriminate analysis techniques. In both negative and positive ionization modes, the tolerance for the precursor and product was set at 5.0 ppm. The peak regions were normalized by IS and subjected to comparison of the detected lipids, with the MainM-score threshold set at 10.0.

### 2.7 mRNA-seq analysis

#### 2.7.1 mRNA isolation and sequencing

Total RNA was extracted from the tissue with TRIzol^®^ reagent according to the manufacturer instructions (Invitrogen), and genomic DNA was removed with DNase I (TaKara). RNA degradation and contamination were observed on 1% agarose gels. The RNA quality was then determined using the 2,100 Bioanalyser (Agilent Technologies) and quantified using the ND-2000 (NanoDrop Technologies). Subsequent library construction and sequencing was entrusted to Mejorbio.

#### 2.7.2 Data analysis

A significance analysis (|fold change| > 1 and *p* value <0.05) was performed to identify genes that were significantly up- or downregulated. After comparing each group to the control group, genes with statistical significance (*p* < 0.05) were selected. The functional annotations of genes used the Kyoto Encyclopedia of Genes and Genomes (KEGG (https://www.kegg.jp). KEGG pathway enrichment analysis was performed using R script and genes/transcripts from the gene set. If the adjusted *p* value (*p* adjust) is less than 0.05, there may be a significant enrichment of this KEGG pathway function. The *q* value was then calculated from the *p* value after several comparisons. The optimized false discovery rate (FDR) approach generated the *q* value list using *p* value distribution characteristics. The grade with a *q* value less than 0.05 (i.e., 5% FDR) was examined.

### 2.8 Primary neuron culture

Primary neurons were obtained from the amygdala of 16–17-day mouse embryos (E16-E17). Neurons were grown at 37°C in medium consisting of neurobasal medium, 1% B27 supplement (Gibco), 2 mM glutamine, and 0.2% Primoin (InvivoGen, FR). Neurons were incubated for 7 days *in vitro* (DIV7) and used for experiments. For mRNA-seq and immunofluorescence assay, the primary neurons were treated with favipiravir for 72 h at a final concentration of 62 or 620 μM. For multielectrode array recording (MEA), the primary neurons were treated with favipiravir for 72 h at a final concentration of 62, 310 or 620 μM (Shannon et al., 2020). The concentrations of DHA-PL liposomes were 5, 10, and 20 μg/mL (Xu et al., 2021), respectively, and the concentration of BEL was 10 μM (Mazzocchi-Jones, 2015). Primary neurons were treated with favipiravir at the presence of DHA-PL liposomes or BEL for 72 h.

### 2.9 Immunofluorescence

The morphology of the primary neuron was investigated with a microscope (Olympus CKX53). For immunostaining, neurons grown on coverslips were fixed with 4% paraformaldehyde for 10 min, permeabilized in PBS with 0.1% Triton X-100 for 10 min and then blocked with 5% bovine serum albumin (BSA). The coverslip was incubated overnight at 4°C with the primary antibody (1:50 to 1:1,000), followed by an incubation with an Alexa Fluor-conjugated secondary antibody (1:200). The antibodies used were listed as follows: anti-TOM20 antibody (11,802–1-AP, Proteintech), anti-Phalloidin antibody (A22287, Invitrogen), and anti-SV2 antibody (AB2315387, Developmental Studies Hybridoma Bank).

Immunofluorescence images were captured with a laser scanning confocal microscope (SP8 X, Leica) equipped with LAS X software. For the quantification of mitochondrial morphology and density, 20 random visual fields were acquired per sample. To ensure fluorescence statistics authenticity, laser intensity in all samples was maintained at a same level. The quantification procedure was performed using ImageJ software (version 1.52P). The line tool was first used to trace the profile of neurites, and the size of the area or the fluorescence intensity of TOM20 or SV2 was calculated. The fluorescence intensity of mitochondria was divided by the corresponding neurite area to obtain the mitochondrial density.

### 2.10 Multielectrode array recording

The MEA system was used to record neuronal electrophysiological activity (Axion BioSystems Inc.). Primary neurons were seeded at a density of 8 × 10^4^ cells per well into poly-D-ornithinecoated 24-well MEA plates with an array of 16 embedded gold electrodes. The neurons were then cultured at 37°C in a humidified incubator with 5% CO_2_. At DIV 7, a pre-recording within 30 min was performed, and then the spontaneous activities of neurons were recorded after favipiravir treatment for 72 h. Raw data files were recorded every 5 min using Axion BioSystems Integrated Studio software (AxIS, version 3.0.2.1). All data were filtered with dual Butterworth filters at 200 Hz (high pass) and 3,000 Hz (low pass). An adaptive threshold spike detector was used on each channel to detect any amplitude greater than or equal to a multiple of six standard deviations (6SD) of the estimated noise. Within 5 min, the number of active electrodes and spikes was recorded. The interspike interval was used to detect bursts (ISI). The AxIS software threshold algorithm has a minimum of 5 peaks per burst and a maximum peak interval of 100 ms.

### 2.11 Statistical analysis

All data were analyzed using GraphPad Prism 8 v8.0.2 (GraphPad Software Inc.) and are presented as the mean ± SD or SEM as indicated in the figure legends. Unpaired two-tailed t tests were used for simple comparisons. For multiple comparisons, Dunnett’s one-way analysis of variance (ANOVA) test was used. For all the results, *, *p* < 0.05; **, *p* < 0.01; ***, *p* < 0.001. All the exact *F* values, *t* values and *p* values for each figure are listed in Supplementary Table 1.

## 3 Results

### 3.1 Favipiravir treatment causes anxiety-like behavior in mice

**FIGURE 1:**
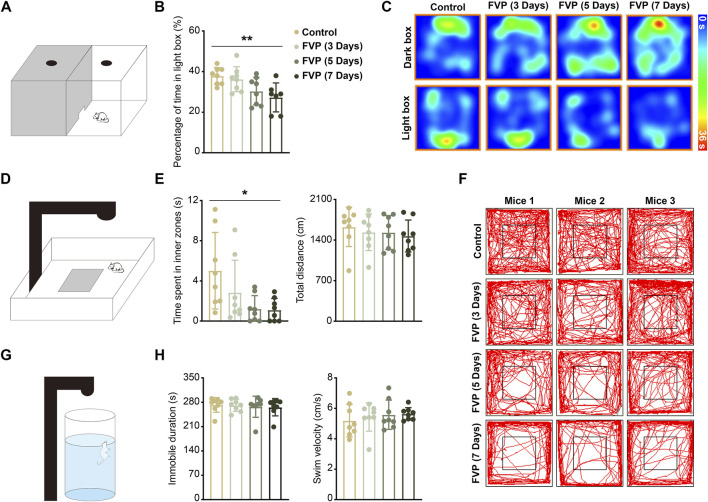
Favipiravir treatment causes anxiety-like behaviors in mice. **(A)** Experimental illustration of the dark/light box test apparatus. **(B)** Percentage of time in the light box of the dark/light box test during 5 min of measurement (*p* = 0.0073). **(C)** Representative heatmaps of each group of mice in the dark/light box test apparatus. **(D)** Experimental illustration of the open field test apparatus. **(E)** Time (seconds) spent in the inner zone (*p* = 0.0206) and distance (cm) traveled in the whole area of the open field test during 5 min of measurement. **(F)** Representative trajectory of each group of mice in the open field test apparatus. The mouse movement path is shown as a red line. **(G)** Experimental illustration of the forced swimming test apparatus. **(H)** Immobility time (seconds) and swim velocity (cm/second) of the forced swimming test during 5 min of measurement. For figure **(B, E, H)**, all data are from eight mice (n = 8). Data are presented as the means ± SDs; *, *p* < 0.05; **, *p* < 0.01; Dunnett’s one-way ANOVA test.

To study whether favipiravir causes anxiety-like behavior, mice were gavaged daily with 200 mg/kg favipiravir for 3, 5, and 7 days, respectively. Light-dark transition open field and forced swimming tests were applied to detect the neurobehaviors of mice. In the light-dark switch test, mice treated with favipiravir exhibited anxiety-like behavior, as evidenced by their significantly reduced time spent in the light chamber compared to the control group. This effect was notably more pronounced after 7 days of favipiravir treatment (*p* < 0.01; Figures 1A–C). Similarly, in the open field test, favipiravir-treated mice displayed an altered pattern of motor activity, characterized by a significant decrease in the time spent in the central area. This effect also intensified with the duration of drug administration, particularly treatment for 7 days (*p* < 0.05; Figures 1D–F). Moreover, favipiravir treatment caused no significant difference in the overall distance traveled by the mice (Figure 1E). We continued to apply the forced swimming test, a chronic stress-inducing task, to investigate behavior in mice. The immobility time and swimming speed were not altered in the mice treated with favipiravir, indicating that favipiravir did not induce depression-like behavior in mice (Figures 1G, H). Collectively, these findings suggested that favipiravir may induce anxiety-like behaviors in mice.

### 3.2 Favipiravir shows no obvious toxic effect in mice

Favipiravir showed no effect on the levels of serum total protein (TP), albumin (ALB), and globulin (GLB) (Supplementary Figure 1A). The levels of serum alkaline phosphatase (ALP) and alanine aminotransferase (ALT) were also unchanged after favipiravir treatment. Favipiravir slightly increased the level of serum aspartate aminotransferase (AST) (Supplementary Figure 1B), which was similar to a previous study (Bayram et al., 2021). Favipiravir treatment caused no changes in the levels of glucose (GLU), urea (UREA), creatinine (CREA), cholesterol (CHOL), and triglyceride (TG) in mice (Supplementary Figure 1C–E). Favipiravir caused no histological changes in the main organs, including the brain, heart, liver, spleen, lung, and kidney (Supplementary Figure 1F).

### 3.3 Favipiravir causes glycerophospholipid dysregulation in the amygdala

**FIGURE 2:**
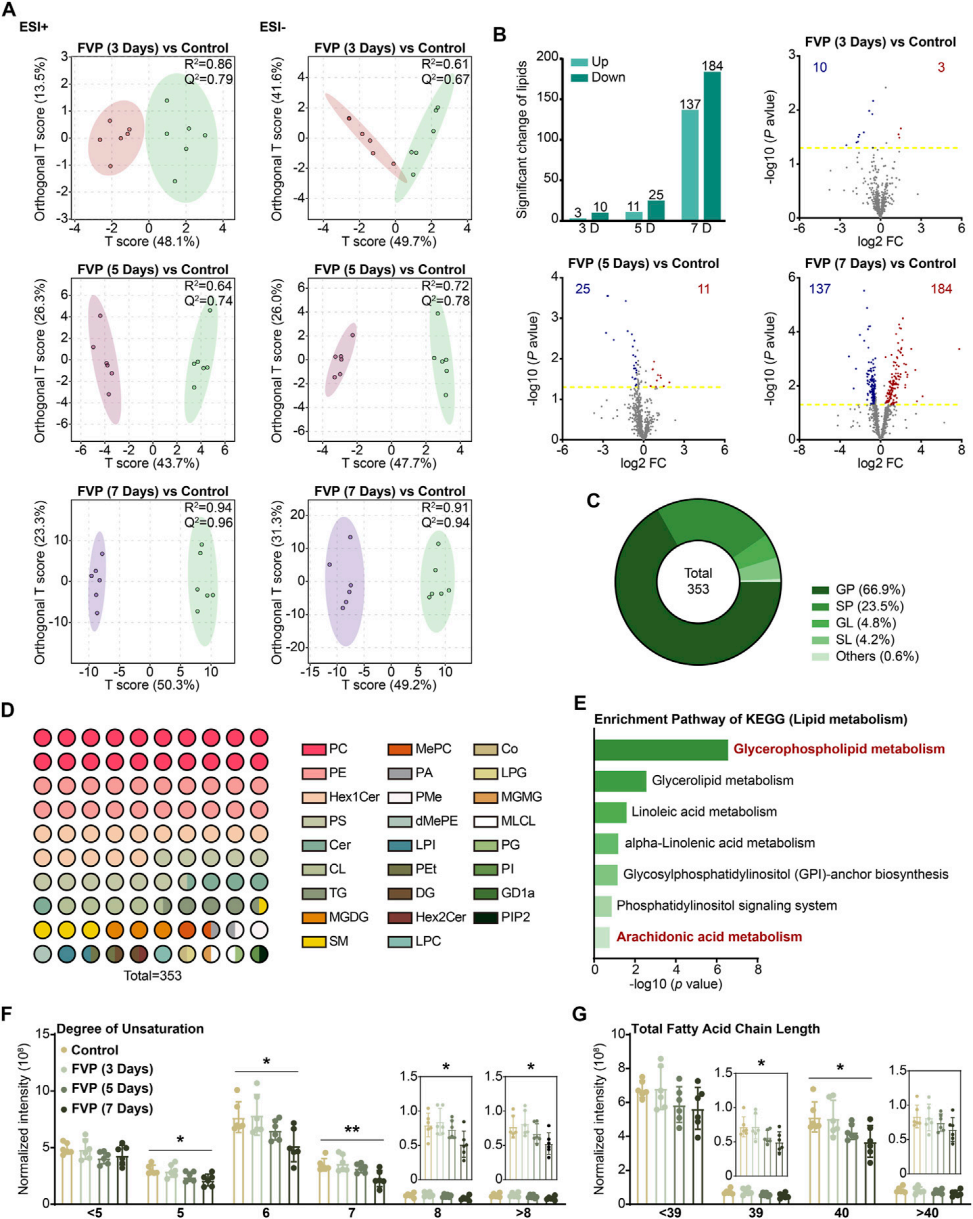
Favipiravir treatment causes significant dysregulation of glycerophospholipid in the amygdala. **(A)** OPLS-DA 2D score scatterplot for the control and favipiravir treatment groups in ESI ± mode. **(B)** Overall significant changes in lipids in the favipiravir treatment groups. Significantly up- or downregulated lipids are marked in red and blue, respectively. **(C)** Pie chart shows the proportion of four major categories lipids (GP, SP, GL, and SL) significantly altered by favipiravir. **(D)** 10 × 10 dot plot shows the proportion of each lipid in all identified lipids. **(E)** The significantly enriched pathways (adjusted *p* < 0.05) in the KEGG pathway analysis of significantly altered lipids by MetaboAnalyst 4.0. **(F, G)** Histograms show the degree of unsaturation (Degree of unsaturation = 5: *p* = 0.0107; Degree of unsaturation = 6: *p* = 0.0131; Degree of unsaturation = 7: *p* = 0.0045; Degree of unsaturation = 8: *p* = 0.0201; Degree of unsaturation>8: *p* = 0.0267) **(F)** and normalized intensity of length (Chain length<39: *p* = 0.0329; Chain length<39: *p* = 0.0269) **(G)** of the acyl chains of all identified GPs. Data from six mice (n = 6). All data are presented as the mean ± SD; *, *p* < 0.05; **, *p* < 0.01; Dunnett’s one-way ANOVA test.

It has been known that the amygdala is linked to generalized anxiety disorder (GAD), and lipid abnormalities in the amygdala are significant drivers of anxiety (Du et al., 2021; Tabbai et al., 2019). We applied lipidomics based on UPLC‒MS-MS in negative and positive ionization modes to investigate the lipidomics of the amygdala in mice treated with favipiravir. Moreover, the orthogonal partial least squares discriminant analysis (OPLS-DA) model was used to identify the different metabolites between the control and favipiravir groups. The results from OPLS-DA in both positive and negative ionization modes showed a complete separation between the control and favipiravir groups. Notably, with the increase in the time of favipiravir treatment, the differences in metabolite aggregation between the two groups were more pronounced, indicating dysregulated lipidomics in the amygdala (Figure 2A). According to the Lipid Map Database (www.lipid-maps.org) and the Human Metabolome Database (https://hmdb.ca/), ion peaks obtained from UPLC‒MS-MS analysis were defined to map different lipid metabolites of the amygdala. Compared with the control group, a total of 353 differential lipid metabolites were identified in the amygdala of mice treated with favipiravir (|log_2_FC(fold change)|>0.5, -log (*p* value) > 1.3) (Figure 2B). With the increase in the administration time of favipiravir, the number of up- and downregulated lipid molecules was significantly increased. When favipiravir was administered for 3 or 5 days continuously, 3 and 11 lipids were upregulated, while 10 and 25 lipids were downregulated, respectively. However, when favipiravir was administered for 7 days, 137 lipids were upregulated and 184 lipids were downregulated (Figure 2B). The significantly altered lipids were classified as glycerophospholipids (GP), sphingolipids (SP), glycerolipids (GL), glycolipids (SL), and isoprenoids (PR), with proportions of 67%, 24%, 5%, and 1%, respectively (Figures 2C, D). We performed pathway enrichment analysis for all significantly altered lipids, and the results showed that those modified lipids were significantly enriched in the GP metabolism pathway (Figure 2E).

The abundance of GP with an unsaturation level of 5 or more decreased dramatically with increasing administration period of favipiravir (Figure 2F). Moreover, the abundance of GP with a chain length of 39 or 40 was significantly decreased (Figure 2G). As the fluidity of neuronal membranes strongly depends on the unsaturation and chain length of GP (Piomelli et al., 2007), these results suggest that favipiravir may decrease membrane fluidity, thus affecting neuronal activity.

### 3.4 Favipiravir reduces the abundance of DHA/AA-PE/PC in the amygdala

**FIGURE 3:**
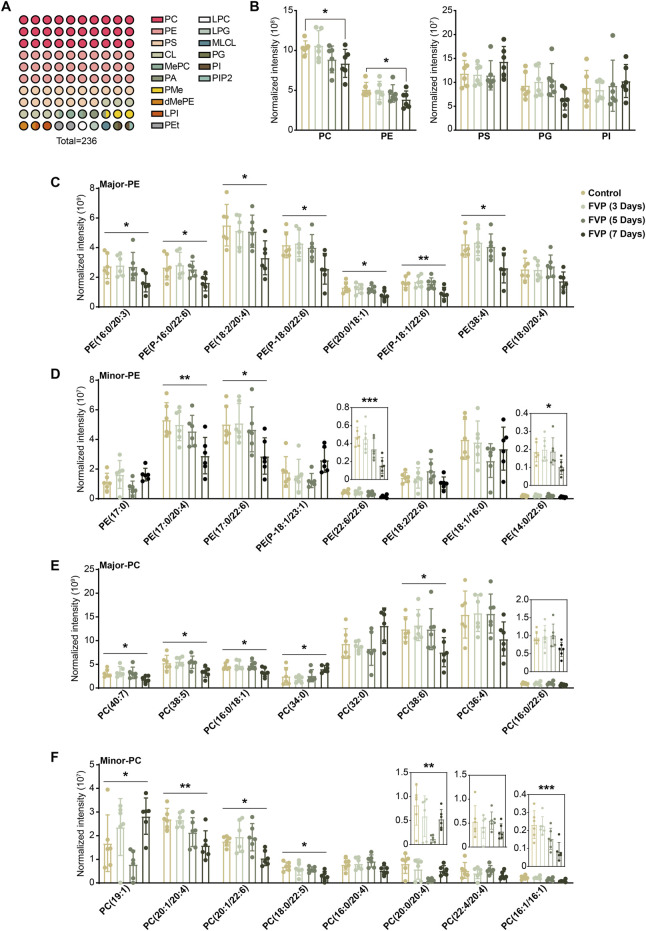
Favipiravir treatment reduces the abundance of DHA/AA-PE/PC in the amygdala. **(A)** A 10 × 10 dot plot shows the proportion of each lipid in all identified GPs. **(B)** Histograms show normalized intensities of PE, PC, PS, PG and PI at different time points after favipiravir treatment. **(C)** Histograms show the normalized intensity of the major species of PE after favipiravir treatment [PE (16:0/20:3): *p* = 0.0238; PE (P-16:0/22:6): *p* = 0.0373; PE (18:0/20:4): *p* = 0.0193; PE (P-18:0/22:6): *p* = 0.0138; PE (20:0/18:1): *p* = 0.0448; PE (P-18:1/22:6): *p* = 0.0062; PE (38:4): *p* = 0.0126]. **(D)** Histograms show the normalized intensity of the minor species of PE after favipiravir treatment [PE (17:0/20:4): *p* = 0.0070; PE (17:0/22:6): *p* = 0.0287; PE (22:6/22:6): *p* = 0.0004; PE (14:0/22:6): *p* = 0.0406]. **(E)** Histograms show the normalized intensity of the major species of PC after favipiravir treatment. [PC(40:7): *p* = 0.0429; PC(38:5): *p* = 0.0147; PC(16:0/18:1): *p* = 0.0265; PC(34:0): *p* = 0.0390; PC(38:6): *p* = 0.0321]. **(F)** Histograms show the normalized intensity of the minor species of PC after favipiravir treatment [PC(19:1): *p* = 0.0101; PC(20:1/20:4): *p* = 0.0032; PC(20:1/22:6): *p* = 0.0129; PC(18:0/22:5): *p* = 0.0122; PC(20:0/20:4): *p* = 0.0089; PC(16:1/16:1): *p* = 0.0010]. Data from six mice (n = 6). All data are presented as the mean ± SD. *, *p* < 0.05; **, *p* < 0.01; ***, *p* < 0.001; Dunnett’s one-way ANOVA test or two-tailed *t*-test.

GP are broadly classified into PC, PE, phosphatidylserine (PS), phosphatidylinositol (PI), phosphatidic acid (PA), phosphatidylglycerol (PG) and cardiolipin (CL). We found that the PE and PC subtypes accounted for approximately 50% of the altered GP and that the overall abundance of PE and PC was steadily reduced by favipiravir (Figures 3A, B). We further examined changes in major or minor PE and PC. The abundance of seven kind of major PE and four kind of minor PE, including six kind of DHA-PE and three kind of AA-PE, was significantly downregulated by favipiravir treatment (Figures 3C, D). In addition, the abundance of four major PC and five minor PC, including one kind of DHA-PC and two kind of AA-PC, decreased with increasing favipiravir administration period (Figures 3E, F). These findings indicated that favipiravir markedly reduced the abundance of DHA-PE/PC and AA-PE/PC in the amygdala. Considering that DHA-PE/PC and AA-PE/PC are associated with neuronal activity (Rashid et al., 2013; Kim et al., 2011), we performed correlation analysis between the changes in DHA-PE/PC and AA-PE/PC abundance with the relative percentage of time in the light box of the dark/light box test or the relative time spent in the inner zone in the open field test. The results showed that decreases in abundance of various DHA-PE/PC and AA-PE/PC were positively correlated with the decrease in the percentage of time in the light box (Supplementary Figure 2); moreover, the decrease in abundance of various DHA-PE/PC and AA-PE/PC were also positively correlated with the decrease in time spent in the inner zone (Supplementary Figure 3). These data suggested that decreases in the abundance of DHA-PE/PC and AA-PE/PC may be related to anxiety-like behavior in mice.

### 3.5 Favipiravir dysregulates the expression of genes associated with arachidonic acid and GP metabolism in the amygdala

**FIGURE 4:**
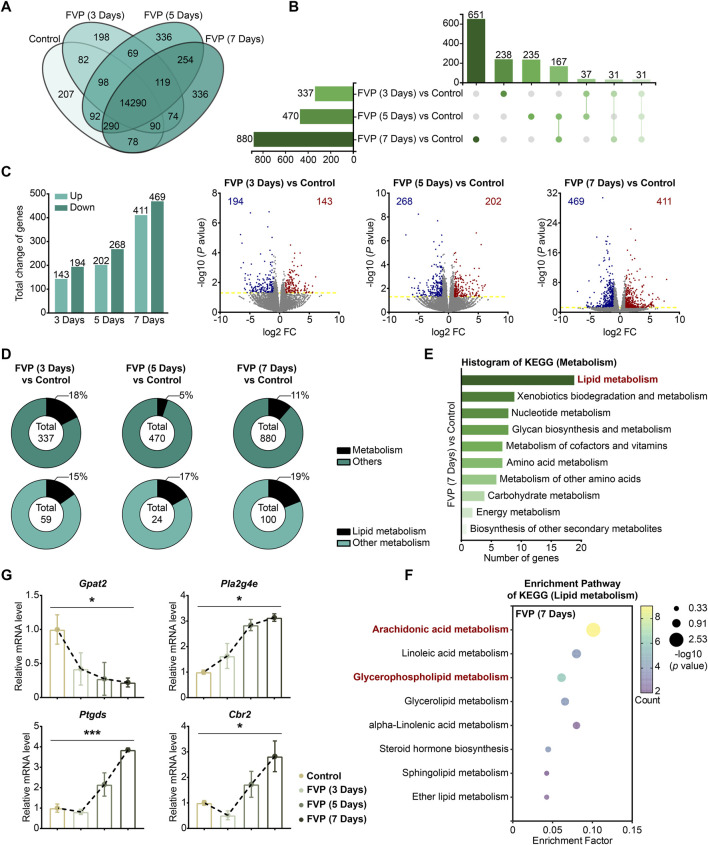
Favipiravir dysregulates the gene expression profile involved in GP metabolism in the amygdala. **(A)** Venn diagrams show the overlapping relationship of all genes between each group. **(B)** UpSet plot showing the number of coexpressed and specifically expressed genes (X-axis) between each group (Y-axis). **(C)** The graph (left) and the number of DEGs in the favipiravir treatment group. The volcano plots (right) show the DEGs marked in red and blue, respectively. **(D)** Proportion of metabolism-related genes in all the detected genes in each group. **(E)** The KEGG pathway functional annotation analysis of the metabolism in the amygdala of mice was shown after favipiravir treatment for 7 days. **(F)** The KEGG pathway functional enrichment analysis of lipid metabolism-related DEGs in the amygdala of mice was shown after favipiravir treatment for 7 days. The ordinate represents the name of the KEGG pathway and the abscissa represents the enrichment factor, the ratio of the number of genes enriched in the KEGG pathway (sample number) to the number of annotated genes (background number). The larger the enrichment factor, the higher the enrichment level. The color of the dot indicates the number of genes enriched in the KEGG pathway, and the size of the dot corresponds to different *p* values. Enrichment results are presented in ascending order of *p* value. **(G)** Relative expression levels of genes modified by favipiravir (*Gpat2*, *Cbr2*, *Pla2g4e*, and *Ptgds*) [*Gpat2*: *p* = 0.0391; *Pla2g4e*: *p* = 0.0336; *Ptgds*: *p* = 0.0005; *Cbr2*: *p* = 0.0180]. Data from three mice (n = 3). All data are presented as the mean ± SEM; *, *p* < 0.05; **, *p* < 0.01; ***, *p* < 0.001; Dunnett’s one-way ANOVA test.

By using an mRNA-seq approach, we analyzed the gene expression profile of the amygdala from mice treated with 200 mg/kg favipiravir for 3, 5 and 7 days. We detected 14,290 genes in all the groups, as shown by the Venn diagram (Figure 4A). The differentially expressed genes (DEGs) were identified among the groups using the criteria log_2_|FC|>1 and *p* < 0.05. We observed that the number of genes significantly altered increased with the increase in favipiravir treatment duration (Figure 4B). In detail, there were 143 upregulated and 194 downregulated genes in the amygdala after favipiravir treatment for 3 days. There were 202 upregulated and 268 downregulated genes after favipiravir treatment for 5 days. After favipiravir treatment for 7 days, we observed 469 upregulated and 411 downregulated genes (Figures 4B, C). Interestingly, among these significantly altered genes, lipid metabolism-related genes involved in metabolic pathways accounted for approximately 20% (Figure 4D). Functional annotation analysis of the KEGG pathway showed that the metabolism-related DEGs were mainly concentrated in the lipid metabolism-related pathways (Supplementary Figure 1A, C, E), especially in the arachidonic acid metabolism pathway after favipiravir treatment for 3 and 5 days (Supplementary Figure 1B, D) as well as in both the arachidonic acid and GP metabolism pathways after favipiravir treatment for 7 days (Figure 4F).

We further analyzed the mRNA expression of the DEGs. The expression of *Gpat2*, a rate-limiting enzyme for GP synthesis (Yamashita et al., 2014), was dramatically downregulated by favipiravir, supporting the reduction in DHA-PE/PC levels mentioned above. The expression of cytoplasmic PLA2 isoforms (*Pla2g4e*, *Pla2g3*) that release AA from phospholipids was also significantly upregulated (Figures 4G, Supplementary Figure 4E). In addition, the expression of genes involved in arachidonic acid metabolism, including prostaglandin D2 synthase (*Ptgds*) and carbonyl reductase 2 (*Cbr2*), was upregulated with prolonged favipiravir treatment (Figure 4G). Considering that abnormally enhanced AA release and metabolism are associated with anxiety (Gaetani et al., 2003), our findings suggested that enhanced AA production may contribute to anxiety-like behavior in favipiravir-treated mice. Taken together, favipiravir caused extensive transcriptomic changes in the amygdala, particularly in the genes related to GP synthesis as well as AA release and metabolism, which may contribute to anxiety-like behaviors in mice.

**FIGURE 5:**
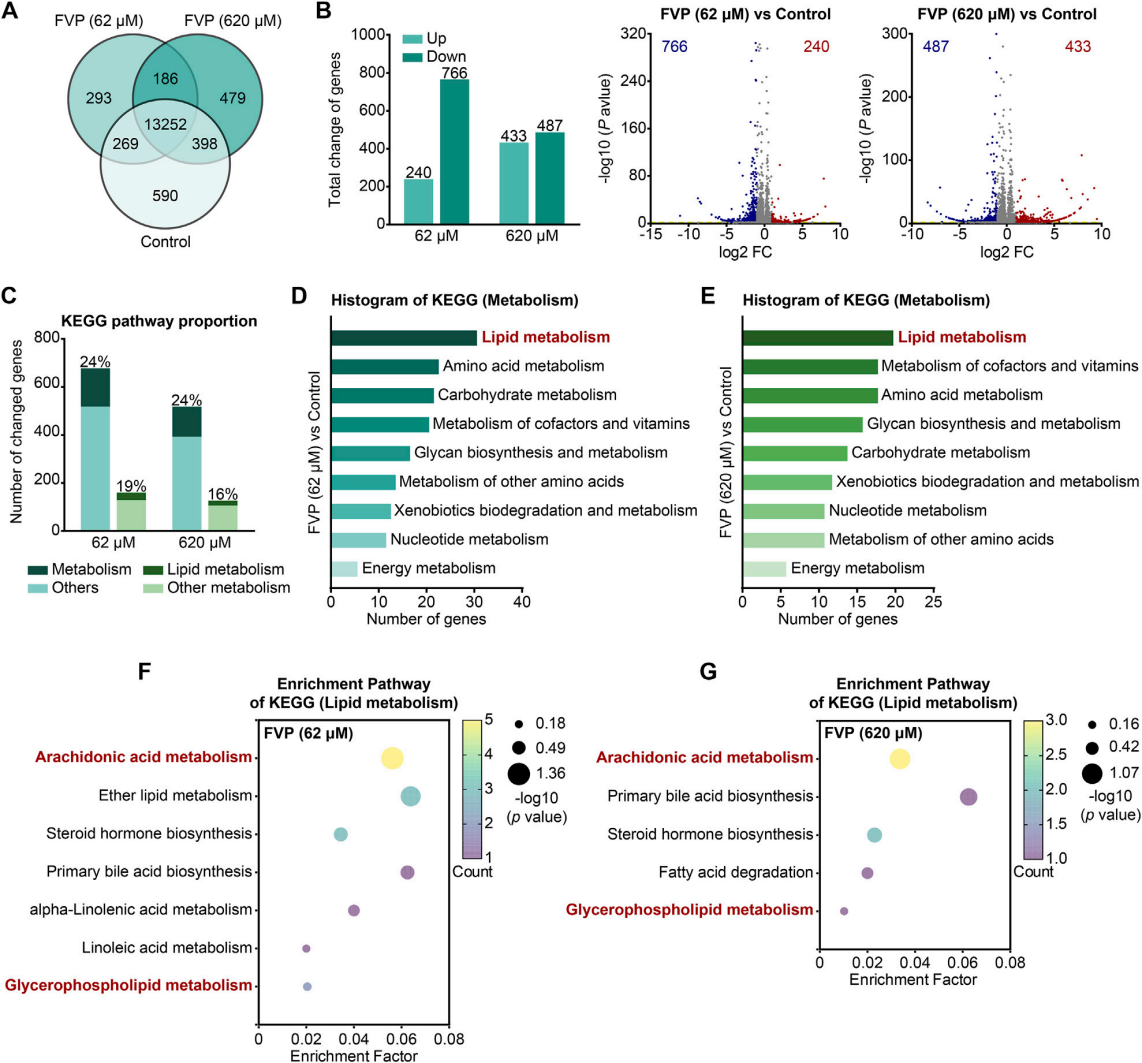
Favipiravir alters the morphology and transcriptome of amygdala neurons. **(A)** Venn diagrams show the overlapping relationship of all genes between each group. **(B)** The graph (left) and the number of DEGs in the favipiravir treatment group in comparison to the control. The volcano plots (right) show the DEGs marked in red and blue, respectively. **(C)** Histogram shows the proportion of metabolism- or lipid metabolism-related genes in all genes or metabolism-related genes, respectively. **(D, E)** The KEGG pathway functional annotation analysis of metabolism related DEGs in primary neurons with favipiravir 62 μM **(D)** or 620 μM **(E)**. The ordinate is the name of the KEGG pathway and the abscissa is the number of genes associated with this pathway. **(F, G)** The KEGG pathway functional enrichment analysis of lipid metabolism-related DEGs in primary neurons with favipiravir 62 μM **(F)** or 620 μM **(G)**.

By mRNA-seq analysis, we further investigated how favipiravir affected the transcriptional profile in primary cultured amygdala neurons *in vitro*. As shown in the Venn diagram, 13,252 genes were detected in all three groups (Figure 5A). The DEGs were identified among the groups using the criteria log_2_|FC|>1 and *p* < 0.05. There were 240 upregulated and 766 downregulated genes after favipiravir (62 μM) treatment for 3 days; there were 433 upregulated and 487 downregulated genes after favipiravir (620 μM) treatment for 3 days (Figure 5B). Among these DEGs, the genes involved in metabolic pathways accounted for approximately 20% and are mainly involved in lipid metabolism (Figure 5C). Furthermore, the KEGG pathway analysis showed that the metabolism-related DEGs in favipiravir treatment groups (62 μM and 620 μM) groups were mainly concentrated in the lipid metabolism pathways (Figures 5D, E), especially in the arachidonic acid and GP metabolism pathways (Figures 5F, G; Supplementary Figure 5). Collectively, these results showed that favipiravir affected the transcriptional profile of primary amygdala neurons *in vitro*, which was consistent with the findings in the amygdala *in vivo*.

### 3.6 Favipiravir alters the morphology and function of amygdala neurons *in vitro*

**FIGURE 6:**
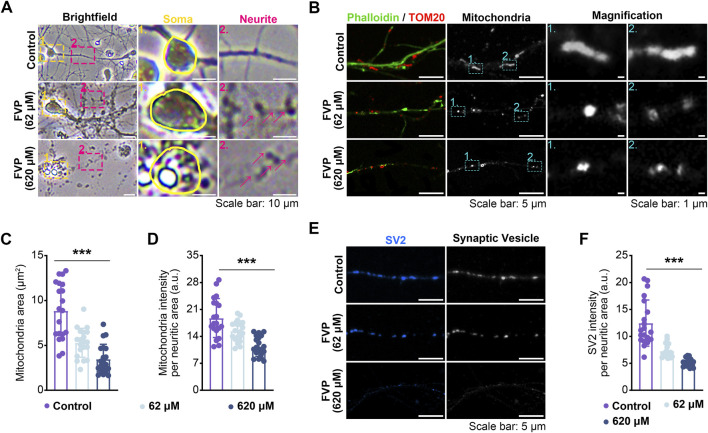
Favipiravir affects the morphology and function of amygdala neurons *in vitro*. **(A)** Bright field images showing the effect of favipiravir on neuronal morphology. **(B)** Fluorescence images show the morphology of mitochondria, and the mitochondrial marker is TOM20. The neurite profile marker is fluorescently labeled phalloidin. **(C, D)** Bar graph shows the mitochondrial area (*p* < 0.0001) **(C)** and intensity per neuritic area (*p* < 0.0001) **(D)**. **(E)** Fluorescence images showing the effect of favipiravir on synaptic vesicles through immunostaining with SV2 protein. **(F)** Bar graph shows SV2 intensity per neuritic area (*p* < 0.0001). At least 20 neurons (n = 20) were chosen for analysis. All quantification data are presented as the mean ± SD; *, *p* < 0.05; **, *p* < 0.01; ***, *p* < 0.001; Dunnett’s one-way ANOVA test.

After the cultured amygdala neurons were treated with favipiravir (62 μM, 620 μM) for 3 days, the morphology, axonal mitochondria, and synaptic vesicles of neurons were examined. Compared with the neurons with thin and smooth axons in the control group, favipiravir-treated neurons showed fragmented structures in the axons (Figure 6A). Since mitochondrial function is closely related to axon morphology and neurobehaviors, including anxiety (Einat et al., 2005), we investigated whether favipiravir affected the mitochondria of axons. Using fluorescently labeled phalloidin and immunostaining of the mitochondrial outer membrane protein TOM20, we observed that the axon mitochondria became shorter and more rounded after favipiravir exposure (62 and 620 μM) for 72 h (Figure 6B); moreover, the area and quantity of mitochondria in neurites were also reduced. These results suggested that favipiravir may disturb the process of mitochondrial fission and fusion in neurons (Figures 6C, D). In addition, favipiravir clearly decreased the intensity of synaptic vesicle protein 2 (SV2), a key neuronal secretory vesicle protein, suggesting disturbance of synaptic vesicle formation and neuronal function (Figures 6E, F). Overall, our results suggest that favipiravir may affect neuronal morphology, axonal mitochondria and synaptic vesicles.

### 3.7 Favipiravir reduces the spontaneous electrical activity of primary amygdala neurons

**FIGURE 7:**
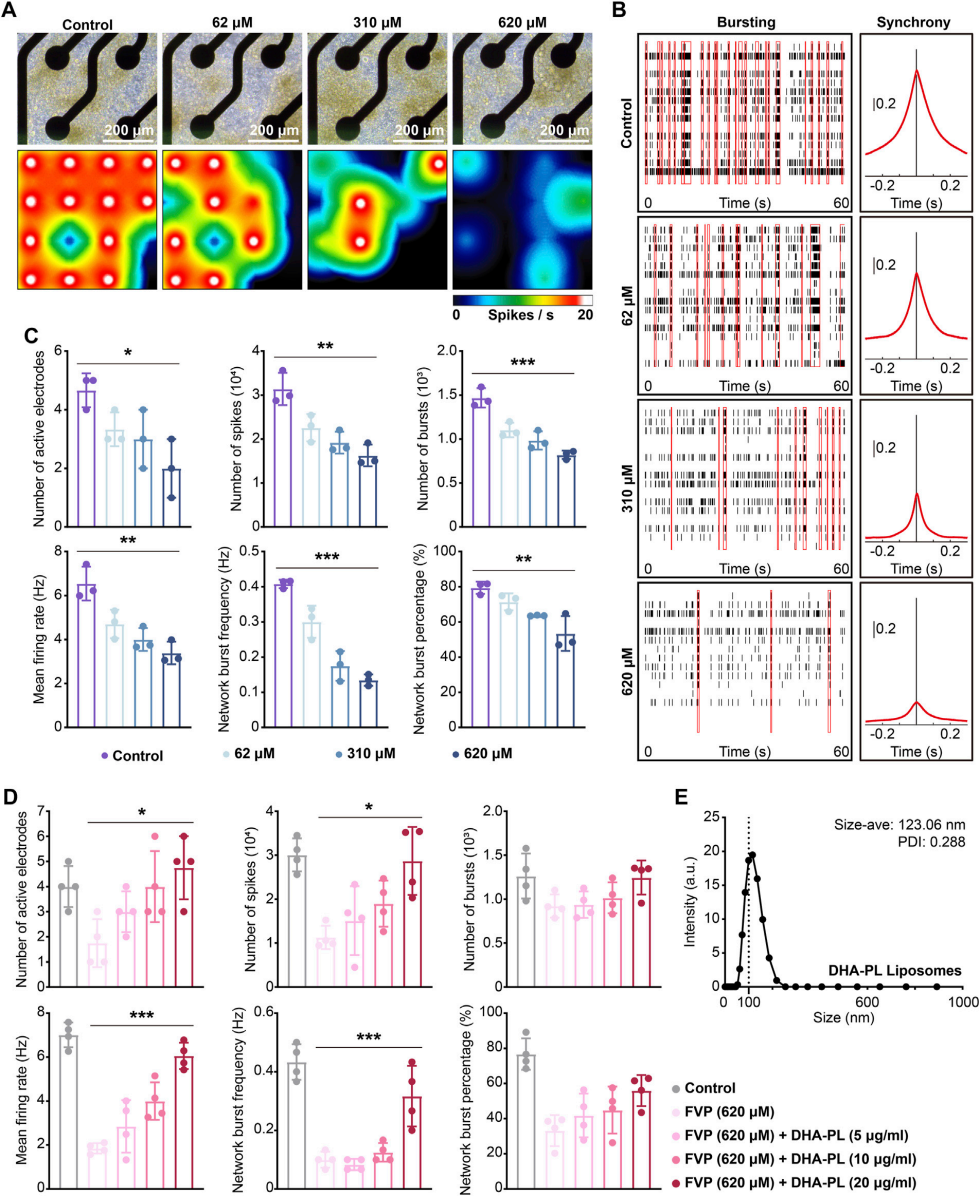
Favipiravir reduces the spontaneous electrical activity of amygdala neurons *in vitro*. **(A)** Right-field images of primary neurons on DIV7 (upper). The heatmap of the representative real-time spike firing rate during MEA recording (lower). The firing rate was color-coded, and the color transitions from white to black. **(B)** Representative raster plots of spikes within 60 s of recording time (left). The area under the cross-correlogram around zero shows the quantification results of synchrony between different electrodes (right). **(C, D)** Individual Favipiravir treatment (number of active electrodes: *p* = 0.0272; number of spikes: *p* = 0.0013; number of bursts: *p* = 0.0001; mean firing rate: *p* = 0.0013; network burst frequency: *p* < 0.0001; network burst percentage: *p* = 0.0031) (or supplementing DHA-PL liposomes (number of active electrodes: *p* = 0.0154; number of spikes: *p* = 0.0113; mean firing rate: *p* < 0.0001; network burst frequency: *p* = 0.0003) **(D)**. Quantification of the number of active electrodes, spikes, bursts, mean firing rate, network burst frequency and percentage during 300 s of recording time. At least 3 wells (n = 3 or n = 4) were chosen for analysis. **(E)** The hydrodynamic of DHA-PL liposomes. All quantification data are presented as the mean ± SD; *, *p* < 0.05; **, *p* < 0.01; ***, *p* < 0.001; Dunnett’s one-way ANOVA test.

We applied MEA to explore the effect of favipiravir on the electrophysiological properties of neurons *in vitro*. After favipiravir exposure (62, 310 and 620 μM) for 72 h, neurons showed a negative trend in the real-time spike firing rate, suggesting a decrease in neuronal viability (Figures 7A, B). Next, we recorded the number of active electrodes, spikes, bursts, average firing rate, network burst frequency and percentage within 300 s. Favipiravir treatment decreased the number of active electrodes, spikes, and bursts, indicating decreases in neuronal excitability and activity (Figure 7C). The average firing rate, network pulse frequency and percentage also decreased dramatically (Figure 7C). Interestingly, when the primary amygdala neurons were supplemented with DHA-PL liposomes (Figure 7E), the number of active electrodes, spikes, average firing rate and network firing frequency, which were reduced by favipiravir, were significantly restored (Figure 7D). Furthermore, supplementation with the PLA2 inhibitor BEL (10 μM) also attenuated the inhibitory effect of favipiravir on the number of active electrodes, bursts, and average firing rate (Supplementary Figure 6A, B). These results suggested that favipiravir may decrease neuronal excitability and network behavior, which may correlate to the anxiety-like behavior caused by favipiravir.

## 4 Discussion

GP is essential components of cell membranes, particularly in neurons, where their composition and function are crucial for neural signal transmission, synaptic plasticity, and cellular health (Frisardi et al., 2011). The amygdala, an important brain area that regulates fear, anxiety, and stress responses (Maldonado et al., 2020). It accepts sensory information and sends it to multiple brain areas, including the hypothalamus, prefrontal cortex, and brainstem (Łoś and Waszkiewicz, 2021). Research has indicated that mice displaying anxiety-like behaviors exhibit abnormal GP metabolism in the brain (Zhou et al., 2024). The lipidomic analysis by Gert Lubec et al. revealed that aged mice exhibited reduced levels of various PE and PC in the amygdala (Šmidák et al., 2017). Similarly, our study observed anxiety-like behaviors in mice following a 7-day treatment with favipiravir. The lipidomics data from our research further revealed that favipiravir significantly impacted GP metabolism in the amygdala.

The changes in the quantity or type of GP, such as acyl chain length and saturation, are able to modify membrane fluidity, thus influencing the function of neurotransmitter receptors, ion channels, and signaling pathways (Shamim et al., 2018; Borroni et al., 2016). Unsaturation of GP in neuronal membranes may affect the function of neurotransmitter systems involved in emotional regulation, thus affecting emotional regulation and cognitive functions (Liu et al., 2018; Falomir-Lockhart et al., 2019; Xia et al., 2020). DHA influences the function of glutamate receptors, including AMPA and NMDA receptors, which are essential for synaptic transmission and neuronal plasticity, by regulating membrane fluidity (Di Miceli et al., 2020; Zhou et al., 2022). Interestingly, we found that favipiravir significantly reduced the unsaturation of GP in the amygdala and decreased levels of various DHA-PE/PC and AA-PE/PC. We hypothesize that favipiravir may alter neuronal membrane fluidity by affecting GP metabolism in the amygdala, thereby disrupting neurotransmitter signaling and neuronal function, which could result in anxiety-like behaviors.

SV2 is a critical component of the synaptic vesicle membrane, essential for the packaging, storage, and release of neurotransmitters (Silm et al., 2019). The effectiveness of SV2 is intricately linked to the membrane fluidity of synaptic vesicles, which is vital for its function and its role in neurotransmitter release (Westra et al., 2021). Our findings indicate that favipiravir administration significantly downregulates SV2, suggesting an inhibition of synaptic vesicle formation. This inhibition may result from a decrease in GP unsaturation and reduced levels of DHA-PE/PC and AA-PE/PC. These changes increase neuronal membrane rigidity, negatively impacting the normal function of synaptic vesicles and subsequently impairing neurotransmitter release.

Mitochondria, known as the cell energy factories, are crucial for ATP production, which is essential for synaptic activity and nerve impulse transmission in neurons (Kann and Kovács, 2007). Their metabolic efficiency and function are closely linked to their morphology, including size, shape, number, and distribution within the cell (López-Doménech and Kittler, 2023). Studies have shown that loss of mitochondria in the basolateral amygdala increases anxiety-related behaviors (Duan et al., 2021) In the social defeat model, mitochondrial size and mass were reduced in the basolateral (BLA) and central (CeA) nuclei of the mouse amygdala (Johnson and Li, 2022). Our study found that favipiravir administration significantly reduced the size and number of mitochondria in amygdala neurons, which may contribute to favipiravir-induced anxiety-like behavior.

It has been known that PC and PE serve as precursors for the synthesis of various neurotransmitters, including acetylcholine and PE-derived neurotransmitters, such as N-acyl ethanolamines (Galkina et al., 2021; Paul et al., 2019). A previous study reported that reduced PE-derived neurotransmitters in the amygdala increase anxiety-like behavior in mice (Bedse et al., 2017; Leishman et al., 2016). In this study, favipiravir significantly reduced the levels of PC and PE, the two major types of GP lipids, in the amygdala. We speculated that the decreased PC and PE may correlate with the aberrant neurobehavior of mice treated with favipiravir. We further defined and clarified the types of PC and PE modified by favipiravir and found that the levels of DHA-PE/PC and AA-PE/PC were decreased dramatically in the amygdala. DHA-PE/PC are involved in signaling and synaptic plasticity, which are crucial for learning and memory (Zhang et al., 2019; Zhang et al., 2020). Moreover, DHA-PE/PC are the main source of DHA for neurons, and they are considered to be the most essential type of DHA in the brain. DHA accounts for more than 90% of the omega 3 polyunsaturated fatty acids (PUFAs) in the brain, mainly as part of the membrane phospholipids in gray matter (Dighriri et al., 2022). When DHA is needed for various biological processes, it can be released from DHA-PE/PC. Our findings indicated that favipiravir decreased the DHA-PE/PC levels in the amygdala of mice. Furthermore, the data from the MEA assay showed that favipiravir dramatically reduced the action potential signaling of neurons *in vitro*, suggesting disturbed neuronal functions. In fact, clinical research has shown that persons with anxiety and depression have lower levels of blood DHA and brain (Parletta et al., 2016). Mice lacking omega-3 PUFAs exhibit disturbed social behavior as well as increased anxiety and depressive behaviors (Larrieu et al., 2016). On the other hand, DHA supplementation improves the cognitive performance of aged rodents (Sun et al., 2018; Pilecky et al., 2021) and alleviates anxiety-like behaviors in male obese rats (Neto et al., 2022). PE/PC are abundant in axons and serve as a key source of AA, a critical mediator of synaptic transmission and intracellular signaling (Yang et al., 2012). AA, an omega-6 fatty acid, stimulates the synthesis of many derivatives associated with depression, and its plasma levels are linked to depression severity (López and Ballaz, 2020). A previous study indicated that AA released from neuronal membranes of the amygdala is linked to anxiety-like behaviors (Morgan et al., 2019).

Acylation of glycerol-3-phosphate (G3P) is the first and most important step in the production of GP, which is catalyzed by the enzyme acyl-CoA: GPAT (Balgoma et al., 2019). Our results showed that favipiravir significantly reduced *Gpat2* expression in the mitochondrial outer membrane. We speculated that such a reduction could result from the decline in PE and PC levels. Similarly, GPAT2 deficiency results in a low level of PC in the brains of mice, which potentially leads to changes in the composition and function of neuronal membranes and results in impaired cognitive performance (Gonzalez-Baro and Coleman, 2017). Loss of GPAT2 causes mitochondrial fragmentation, leading to abnormal function (Ohba et al., 2013). We speculate that downregulated *Gpat2* may correlate with the fragmented mitochondria in neurons. Further research is needed to address this issue.

As expected, mRNA sequencing analysis also showed that the expression of cytosolic *Pla2* isoforms was increased dramatically in the amygdala after favipiravir treatment. PLA2 releases arachidonic acid from membrane phospholipids (Aizawa et al., 2016), and enhanced expression of PLA2 reflects active inflammatory responses and anxiety-like behavior (Furuyashiki et al., 2019). In contrast, administration of PLA2 inhibitors can alleviate anxiety-like behavior in rats (Farooqui et al., 2006). In addition, abnormal activation of PLA2 and subsequent release of AA can promote mitochondrial membrane permeabilization, leading to mitochondrial stress (Giovannoni and Quintana, 2020). This may explain the fragmented mitochondria and disturbed neuronal firing function observed in the neurons treated with favipiravir in the present study.

In conclusion, We combined mass spectrometry-based lipidomics and mRNA-seq analysis to investigate the mechanisms of favipiravir-induced anxiety-like behaviors. Our findings revealed altered GP metabolism in the amygdala, which displayed a remarkable reduction in the abundance of DHA/AA-PE/PC as well as unsaturation of GPs. Favipiravir reduced the mRNA expression of *Gpat2* while increased the expression of *Pla2* isoforms involved in AA release. Furthermore, favipiravir altered neurite morphology and the structure of axonal mitochondria and reduced synaptic vesicles, which may correlate with dysregulated neurophysiological activity. Taken above, we propose that dysregulated GP metabolism in the amygdala may attribute to anxiety-like behavior caused by favipiravir.

## Data Availability

The original contributions presented in the study are publicly available. This data can be found here: NCBI repository, accession numbers PRJNA1227588, PRJNA1228171.

## References

[B1] Aizawa F. Nishinaka T. Yamashita T. Nakamoto K. Koyama Y. Kasuya F. (2016). Astrocytes release polyunsaturated fatty acids by lipopolysaccharide stimuli. Biol. Pharm. Bull. 39 (7), 1100–1106. 10.1248/bpb.b15-01037 27374285

[B2] Aktaş A. Tüzün B. Aslan R. Sayin K. Ataseven H. (2021). New anti-viral drugs for the treatment of covid-19 instead of favipiravir. J. Biomol. Struct. Dyn. 39 (18), 7263–7273. 10.1080/07391102.2020.1806112 32783586 PMC7484583

[B3] Aldana J. Romero-Otero A. Cala M. P. (2020). Exploring the lipidome: current lipid extraction techniques for mass spectrometry analysis. Metabolites 10 (6), 231. 10.3390/metabo10060231 32503331 PMC7345237

[B4] Balgoma D. Pettersson C. Hedeland M. (2019). Common fatty markers in diseases with dysregulated lipogenesis. Trends Endocrinol. and Metabolism 30 (5), 283–285. 10.1016/j.tem.2019.02.008 30926249

[B5] Bayram M. Yildirim O. Ozmen R. S. Soylu B. Dundar A. S. Koksal A. R. (2021). Elevation of serum transaminase levels due to favipiravir use in the treatment of Covid-19. Cureus 13 (9), e18166. 10.7759/cureus.18166 34703696 PMC8530246

[B6] Bedse G. Hartley N. D. Neale E. Gaulden A. D. Patrick T. A. Kingsley P. J. (2017). Functional redundancy between canonical endocannabinoid signaling systems in the modulation of anxiety. Biol. Psychiatry 82 (7), 488–499. 10.1016/j.biopsych.2017.03.002 28438413 PMC5585044

[B7] Borroni M. V. Vallés A. S. Barrantes F. J. (2016). The lipid habitats of neurotransmitter receptors in brain. Biochimica Biophysica Acta (BBA) - Biomembr. 1858 (11), 2662–2670. 10.1016/j.bbamem.2016.07.005 27424801

[B8] Chen C. Huang J. Yin P. Zhang Y. Cheng Z. Wu J. (2020). Favipiravir versus arabitol for covid-19: a randomized clinical trial. MedRxiv, 2020:2020.2003. 2017.20037432.

[B9] Chen C. Zhang Y. Huang J. Yin P. Cheng Z. Wu J. (2021). Favipiravir versus arbidol for clinical recovery rate in moderate and severe adult covid-19 patients: a prospective, multicenter, open-label, randomized controlled clinical trial. Front. Pharmacol. 12, 683296. 10.3389/fphar.2021.683296 34539392 PMC8443786

[B10] Chiurchiù V. Maccarrone M. (2016). Bioactive lipids as modulators of immunity, inflammation and emotions. Curr. Opin. Pharmacol. 29, 54–62. 10.1016/j.coph.2016.06.005 27372887

[B11] Coker C. R. Keller B. N. Arnold A. C. Silberman Y. (2021). Impact of high fat diet and ethanol consumption on neurocircuitry regulating emotional processing and metabolic function. Front. Behav. Neurosci. 14, 601111. 10.3389/fnbeh.2020.601111 33574742 PMC7870708

[B12] De Clercq E. (2019). New nucleoside analogues for the treatment of hemorrhagic fever virus infections. Chem. Asian J. 14 (22), 3962–3968. 10.1002/asia.201900841 31389664 PMC7159701

[B13] Dighriri I. M. Alsubaie A. M. Hakami F. M. Hamithi D. M. Alshekh M. M. Khobrani F. A. (2022). Effects of omega-3 polyunsaturated fatty acids on brain functions: a systematic review. Cureus 14 (10), e30091. 10.7759/cureus.30091 36381743 PMC9641984

[B14] Di Miceli M. Bosch-Bouju C. Layé S. (2020). Pufa and their derivatives in neurotransmission and synapses: a new hallmark of synaptopathies. Proc. Nutr. Soc. 79 (4), 388–403. 10.1017/s0029665120000129 32299516

[B15] Du Y. Li H. Xiao H. Wang M. Zhang W. Gong Q. (2021). Illness severity moderated association between trait anxiety and amygdala-based functional connectivity in generalized anxiety disorder. Front. Behav. Neurosci. 15, 637426. 10.3389/fnbeh.2021.637426 33867949 PMC8044966

[B16] Duan K. Gu Q. Petralia R. S. Wang Y.-X. Panja D. Liu X. (2021). Mitophagy in the basolateral amygdala mediates increased anxiety induced by aversive social experience. Neuron 109 (23), 3793–3809.e8. 10.1016/j.neuron.2021.09.008 34614419 PMC8639775

[B17] Duyan M. Ozturan I. U. (2021). Acute psychosis in covid-19: is it due to favipiravir treatment or acute viral illness? SN Compr. Clin. Med. 3 (7), 1627–1629. 10.1007/s42399-021-00952-x 33997622 PMC8106544

[B18] Einat H. Yuan P. Manji H. K. (2005). Increased anxiety-like behaviors and mitochondrial dysfunction in mice with targeted mutation of the bcl-2 gene: further support for the involvement of mitochondrial function in anxiety disorders. Behav. Brain Res. 165 (2), 172–180. 10.1016/j.bbr.2005.06.012 16095731

[B19] Falomir-Lockhart L. J. Cavazzutti G. F. Giménez E. Toscani A. M. (2019). Fatty acid signaling mechanisms in neural cells: fatty acid receptors. Front. Cell. Neurosci. 13, 162. 10.3389/fncel.2019.00162 31105530 PMC6491900

[B20] Farooqui A. A. Ong W.-Y. Horrocks L. A. (2006). Inhibitors of brain phospholipase a2 activity: their neuropharmacological effects and therapeutic importance for the treatment of neurologic disorders. Pharmacol. Rev. 58 (3), 591–620. 10.1124/pr.58.3.7 16968951

[B21] Frisardi V. Panza F. Seripa D. Farooqui T. Farooqui A. A. (2011). Glycerophospholipids and glycerophospholipid-derived lipid mediators: a complex meshwork in alzheimer’s disease pathology. Prog. lipid Res. 50 (4), 313–330. 10.1016/j.plipres.2011.06.001 21703303

[B22] Furuta Y. Komeno T. Nakamura T. (2017). Favipiravir (t-705), a broad spectrum inhibitor of viral rna polymerase. Proc. Jpn. Acad. Ser. B Phys. Biol. Sci. 93 (7), 449–463. 10.2183/pjab.93.027 PMC5713175 28769016

[B23] Furuyashiki T. Akiyama S. Kitaoka S. (2019). Roles of multiple lipid mediators in stress and depression. Int. Immunol. 31 (9), 579–587. 10.1093/intimm/dxz023 30810163

[B24] Gaetani S. Cuomo V. Piomelli D. (2003). Anandamide hydrolysis: a new target for anti-anxiety drugs? Trends Mol. Med. 9 (11), 474–478. 10.1016/j.molmed.2003.09.005 14604824

[B25] Galkina O. Vetrovoy O. Eschenko N. (2021). The role of lipids in implementing specific functions in the central nervous system. Russ. J. Bioorg. Chem. 47, 1004–1013. 10.1134/s1068162021050253

[B26] Garcia Corrales A. V. Haidar M. Bogie J. F. J. Hendriks J. J. A. (2021). Fatty acid synthesis in glial cells of the cns. Int. J. Mol. Sci. 22 (15), 8159. 10.3390/ijms22158159 34360931 PMC8348209

[B27] Giovannoni F. Quintana F. J. (2020). The role of astrocytes in cns inflammation. Trends Immunol. 41 (9), 805–819. 10.1016/j.it.2020.07.007 32800705 PMC8284746

[B28] Gonzalez-Baro M. R. Coleman R. A. (2017). Mitochondrial acyltransferases and glycerophospholipid metabolism. Biochimica Biophysica Acta (BBA)-Molecular Cell Biol. Lipids 1862 (1), 49–55. 10.1016/j.bbalip.2016.06.023 27377347

[B29] Gunduz-Cinar O. Hill M. N. Mcewen B. S. Holmes A. (2013). Amygdala faah and anandamide: mediating protection and recovery from stress. Trends Pharmacol. Sci. 34 (11), 637–644. 10.1016/j.tips.2013.08.008 24325918 PMC4169112

[B30] Hassanipour S. Arab-Zozani M. Amani B. Heidarzad F. Fathalipour M. Martinez-De-Hoyo R. (2021). The efficacy and safety of favipiravir in treatment of covid-19: a systematic review and meta-analysis of clinical trials. Sci. Rep. 11 (1), 11022. 10.1038/s41598-021-90551-6 34040117 PMC8155021

[B31] Hossain Z. Kurihara H. Hosokawa M. Takahashi K. (2006). Docosahexaenoic acid and eicosapentaenoic acid-enriched phosphatidylcholine liposomes enhance the permeability, transportation and uptake of phospholipids in caco-2 cells. Mol. Cell. Biochem. 285 (1), 155–163. 10.1007/s11010-005-9074-6 16477371

[B32] Hu P. Lu Y. Pan B. X. Zhang W. H. (2022). New insights into the pivotal role of the amygdala in inflammation-related depression and anxiety disorder. Int. J. Mol. Sci. 23 (19), 11076. 10.3390/ijms231911076 36232376 PMC9570160

[B33] Johnson J. Li Z. (2022). Fuel for emotion: how mitophagy in the bla can mediate increased anxiety in stressful social situations. Autophagy 18 (2), 465–466. 10.1080/15548627.2021.2014769 34927540 PMC8942539

[B34] Kann O. Kovács R. (2007). Mitochondria and neuronal activity. Am. J. Physiology-Cell Physiology 292 (2), C641–C657. 10.1152/ajpcell.00222.2006 17092996

[B35] Kao Y. C. Ho P. C. Tu Y. K. Jou I. M. Tsai K. J. (2020). Lipids and alzheimer's disease. Int. J. Mol. Sci. 21 (4), 1505. 10.3390/ijms21041505 32098382 PMC7073164

[B36] Kaur R. J. Charan J. Dutta S. Sharma P. Bhardwaj P. Sharma P. (2020). Favipiravir use in covid-19: analysis of suspected adverse drug events reported in the who database. Infect. Drug Resist 13, 4427–4438. 10.2147/IDR.S287934 33364790 PMC7751706

[B37] Kim H.-Y. Moon H.-S. Cao D. Lee J. Kevala K. Jun S. B. (2011). N-docosahexaenoylethanolamide promotes development of hippocampal neurons. Biochem. J. 435 (2), 327–336. 10.1042/BJ20102118 21281269 PMC3169088

[B38] Larrieu T. Hilal M. L. Smedt-Peyrusse D. Sans N. Layé S. (2016). Nutritional omega-3 deficiency alters glucocorticoid receptor-signaling pathway and neuronal morphology in regionally distinct brain structures associated with emotional deficits. Neural plast. 2016, 8574830. 10.1155/2016/8574830 27057368 PMC4710953

[B39] Leishman E. Cornett B. Spork K. Straiker A. Mackie K. Bradshaw H. B. (2016). Broad impact of deleting endogenous cannabinoid hydrolyzing enzymes and the cb1 cannabinoid receptor on the endogenous cannabinoid-related lipidome in eight regions of the mouse brain. Pharmacol. Res. 110, 159–172. 10.1016/j.phrs.2016.04.020 27109320 PMC4914450

[B40] Liu F. Wang C. Slikker W. (2021). Analysis of biofluid lipid changes: potential biomarkers for detecting central nervous system diseases and neurotoxicity. Curr. Opin. Toxicol. 28, 15–19. 10.1016/j.cotox.2021.08.008

[B41] Liu J. J. Hezghia A. Shaikh S. R. Cenido J. F. Stark R. E. Mann J. J. (2018). Regulation of monoamine transporters and receptors by lipid microdomains: implications for depression. Neuropsychopharmacology 43 (11), 2165–2179. 10.1038/s41386-018-0133-6 30022062 PMC6135777

[B42] López D. E. Ballaz S. J. (2020). The role of brain cyclooxygenase-2 (cox-2) beyond neuroinflammation: neuronal homeostasis in memory and anxiety. Mol. Neurobiol. 57 (12), 5167–5176. 10.1007/s12035-020-02087-x 32860157

[B43] López-Doménech G. Kittler J. T. (2023). Mitochondrial regulation of local supply of energy in neurons. Curr. Opin. Neurobiol. 81, 102747. 10.1016/j.conb.2023.102747 37392672 PMC11139648

[B44] Łoś K. Waszkiewicz N. (2021). Biological markers in anxiety disorders. J. Clin. Med. 10 (8), 1744. 10.3390/jcm10081744 33920547 PMC8073190

[B45] Madelain V. Mentré F. Baize S. Anglaret X. Laouénan C. Oestereich L. (2020b). Modeling favipiravir antiviral efficacy against emerging viruses: from animal studies to clinical trials. CPT pharmacometrics and Syst. Pharmacol. 9 (5), 258–271. 10.1002/psp4.12510 PMC7239338 32198838

[B46] Madelain V. Mentré F. Baize S. Anglaret X. Laouénan C. Oestereich L. (2020a). Modeling favipiravir antiviral efficacy against emerging viruses: from animal studies to clinical trials. CPT Pharmacometrics and Syst. Pharmacol. 9 (5), 258–271. 10.1002/psp4.12510 PMC7239338 32198838

[B47] Maldonado R. Cabañero D. Martín-García E. (2020). The endocannabinoid system in modulating fear, anxiety, and stress. Dialogues Clin. Neurosci. 22 (3), 229–239. 10.31887/DCNS.2020.22.3/rmaldonado 33162766 PMC7605023

[B48] Mazzocchi-Jones D. (2015). Impaired corticostriatal ltp and depotentiation following ipla2 inhibition is restored following acute application of dha. Brain Res. Bull. 111, 69–75. 10.1016/j.brainresbull.2014.12.010 25562715

[B49] Morgan A. Kondev V. Bedse G. Baldi R. Marcus D. Patel S. (2019). Cyclooxygenase-2 inhibition reduces anxiety-like behavior and normalizes enhanced amygdala glutamatergic transmission following chronic oral corticosterone treatment. Neurobiol. Stress 11, 100190. 10.1016/j.ynstr.2019.100190 31467944 PMC6710559

[B50] Neto J. Jantsch J. De Oliveira S. Braga M. F. Castro L. F. D. S. Diniz B. F. (2022). Dha/epa supplementation decreases anxiety-like behaviour, but it does not ameliorate metabolic profile in obese male rats. Br. J. Nutr. 128 (5), 964–974. 10.1017/S0007114521003998 34605386

[B51] Ohba Y. Sakuragi T. Kage-Nakadai E. Tomioka N. H. Kono N. Imae R. (2013). Mitochondria-type gpat is required for mitochondrial fusion. Embo J. 32 (9), 1265–1279. 10.1038/emboj.2013.77 23572076 PMC3642685

[B52] Parletta N. Zarnowiecki D. Cho J. Wilson A. Procter N. Gordon A. (2016). People with schizophrenia and depression have a low omega-3 index. Prostagl. Leukot. Essent. Fat. Acids 110, 42–47. 10.1016/j.plefa.2016.05.007 27255642

[B53] Paul S. Lancaster G. I. Meikle P. J. (2019). Plasmalogens: a potential therapeutic target for neurodegenerative and cardiometabolic disease. Prog. lipid Res. 74, 186–195. 10.1016/j.plipres.2019.04.003 30974122

[B54] Pilecky M. Závorka L. Arts M. T. Kainz M. J. (2021). Omega‐3 pufa profoundly affect neural, physiological, and behavioural competences–implications for systemic changes in trophic interactions. Biol. Rev. 96 (5), 2127–2145. 10.1111/brv.12747 34018324

[B55] Piomelli D. Astarita G. Rapaka R. (2007). A neuroscientist's guide to lipidomics. Nat. Rev. Neurosci. 8 (10), 743–754. 10.1038/nrn2233 17882252

[B56] Rashid M. A. Katakura M. Kharebava G. Kevala K. Kim H.-Y. (2013). N-docosahexaenoylethanolamine is a potent neurogenic factor for neural stem cell differentiation. J. Neurochem. 125 (6), 869–884. 10.1111/jnc.12255 23570577 PMC3775276

[B57] Raulin A. C. Martens Y. A. Bu G. (2022). Lipoproteins in the central nervous system: from biology to pathobiology. Annu. Rev. Biochem. 91, 731–759. 10.1146/annurev-biochem-032620-104801 35303786 PMC9634960

[B58] Shah P. L. Orton C. M. Grinsztejn B. Donaldson G. C. Ramírez B. C. Tonkin J. (2022). Favipiravir in patients hospitalised with covid-19 (pioneer trial): a multicentre, open-label, phase 3, randomised controlled trial of early intervention versus standard care. Lancet Respir. Med. 11, 415–424. 10.1016/S2213-2600(22)00412-X 36528039 PMC9891737

[B59] Shamim A. Mahmood T. Ahsan F. Kumar A. Bagga P. (2018). Lipids: an insight into the neurodegenerative disorders. Clin. Nutr. Exp. 20, 1–19. 10.1016/j.yclnex.2018.05.001

[B60] Shannon A. Selisko B. Le N.-T.-T. Huchting J. Touret F. Piorkowski G. (2020). Rapid incorporation of favipiravir by the fast and permissive viral rna polymerase complex results in sars-cov-2 lethal mutagenesis. Nat. Commun. 11 (1), 4682. 10.1038/s41467-020-18463-z 32943628 PMC7499305

[B61] Shinohara M. Tachibana M. Kanekiyo T. Bu G. (2017). Role of lrp1 in the pathogenesis of alzheimer's disease: evidence from clinical and preclinical studies. J. Lipid Res. 58 (7), 1267–1281. 10.1194/jlr.R075796 28381441 PMC5496044

[B62] Shrestha D. B. Budhathoki P. Khadka S. Shah P. B. Pokharel N. Rashmi P. (2020). Favipiravir versus other antiviral or standard of care for covid-19 treatment: a rapid systematic review and meta-analysis. Virol. J. 17 (1), 141. 10.1186/s12985-020-01412-z 32972430 PMC7512218

[B63] Sienski G. Narayan P. Bonner J. M. Kory N. Boland S. Arczewska A. A. (2021). Apoe4 disrupts intracellular lipid homeostasis in human ipsc-derived glia. Sci. Transl. Med. 13 (583), eaaz4564. 10.1126/scitranslmed.aaz4564 33658354 PMC8218593

[B64] Silm K. Yang J. Marcott P. F. Asensio C. S. Eriksen J. Guthrie D. A. (2019). Synaptic vesicle recycling pathway determines neurotransmitter content and release properties. Neuron 102 (4), 786–800.e5. 10.1016/j.neuron.2019.03.031 31003725 PMC6541489

[B65] Šmidák R. Köfeler H. C. Hoeger H. Lubec G. (2017). Comprehensive identification of age-related lipidome changes in rat amygdala during normal aging. PLoS One 12 (7), e0180675. 10.1371/journal.pone.0180675 28672041 PMC5495493

[B66] Solaymani-Dodaran M. Ghanei M. Bagheri M. Qazvini A. Vahedi E. Hassan Saadat S. (2021). Safety and efficacy of favipiravir in moderate to severe sars-cov-2 pneumonia. Int. Immunopharmacol. 95, 107522. 10.1016/j.intimp.2021.107522 33735712 PMC7951885

[B67] Steen V. M. Skrede S. Polushina T. López M. Andreassen O. A. Fernø J. (2017). Genetic evidence for a role of the srebp transcription system and lipid biosynthesis in schizophrenia and antipsychotic treatment. Eur. Neuropsychopharmacol. 27 (6), 589–598. 10.1016/j.euroneuro.2016.07.011 27492885

[B68] Sun G. Y. Simonyi A. Fritsche K. L. Chuang D. Y. Hannink M. Gu Z. (2018). Docosahexaenoic acid (dha): an essential nutrient and a nutraceutical for brain health and diseases. Prostagl. Leukot. Essent. Fat. Acids 136, 3–13. 10.1016/j.plefa.2017.03.006 PMC9087135 28314621

[B69] Suzuki M. Masuda Y. (2008). Effect of a neuraminidase inhibitor (oseltamivir) on mouse jump-down behavior via stimulation of dopamine receptors. Biomed. Res. 29 (5), 233–238. 10.2220/biomedres.29.233 18997437

[B70] Tabbai S. Moreno-Fernández R. D. Zambrana-Infantes E. Nieto-Quero A. Chun J. García-Fernández M. (2019). Effects of the lpa(1) receptor deficiency and stress on the hippocampal lpa species in mice. Front. Mol. Neurosci. 12, 146. 10.3389/fnmol.2019.00146 31244601 PMC6580287

[B71] Tani H. Komeno T. Fukuma A. Fukushi S. Taniguchi S. Shimojima M. (2018). Therapeutic effects of favipiravir against severe fever with thrombocytopenia syndrome virus infection in a lethal mouse model: dose-efficacy studies upon oral administration. PloS one 13 (10), e0206416. 10.1371/journal.pone.0206416 30365543 PMC6203377

[B72] Ueda M. Tanimoto T. Murayama A. Ozaki A. Kami M. (2022). Japan’s drug regulation during the covid‐19 pandemic: lessons from a case study of favipiravir. Clin. Pharmacol. Ther. 111 (3), 545–547. 10.1002/cpt.2251 33882157 PMC8251038

[B73] Wang C. Liu F. Frisch-Daiello J. L. Martin S. Patterson T. A. Gu Q. (2018). Lipidomics reveals a systemic energy deficient state that precedes neurotoxicity in neonatal monkeys after sevoflurane exposure. Anal. Chim. Acta 1037, 87–96. 10.1016/j.aca.2017.11.052 30292318

[B74] Wang X. Wu F. Zou H. Yang Y. Chen G. Liu K. (2022). Neurodevelopmental toxicity of pyrazinamide to larval zebrafish and the restoration after intoxication withdrawal. J. Appl. Toxicol. 42 (7), 1276–1286. 10.1002/jat.4294 35102572

[B75] Westra M. Gutierrez Y. Macgillavry H. D. (2021). Contribution of membrane lipids to postsynaptic protein organization. Front. Synaptic Neurosci. 13, 790773. 10.3389/fnsyn.2021.790773 34887741 PMC8649999

[B76] Xia W. Liu G. Shao Z. Xu E. Yuan H. Liu J. (2020). Toxicology of tramadol following chronic exposure based on metabolomics of the cerebrum in mice. Sci. Rep. 10 (1), 11130–11211. 10.1038/s41598-020-67974-8 32636435 PMC7341866

[B77] Xu Z. J. Li Q. Ding L. Shi H. H. Xue C. H. Mao X. Z. (2021). A comparative study of the effects of phosphatidylserine rich in dha and epa on aβ-induced alzheimer's disease using cell models. Food Funct. 12 (10), 4411–4423. 10.1039/d1fo00286d 33876786

[B78] Yamashita A. Hayashi Y. Matsumoto N. Nemoto-Sasaki Y. Oka S. Tanikawa T. (2014). Glycerophosphate/acylglycerophosphate acyltransferases. Biology 3 (4), 801–830. 10.3390/biology3040801 25415055 PMC4280512

[B79] Yang H.-J. Sugiura Y. Ikegami K. Konishi Y. Setou M. (2012). Axonal gradient of arachidonic acid-containing phosphatidylcholine and its dependence on actin dynamics. J. Biol. Chem. 287 (8), 5290–5300. 10.1074/jbc.M111.316877 22207757 PMC3285309

[B80] Yu H. Villanueva N. Bittar T. Arsenault E. Labonté B. Huan T. (2020). Parallel metabolomics and lipidomics enables the comprehensive study of mouse brain regional metabolite and lipid patterns. Anal. Chim. Acta 1136, 168–177. 10.1016/j.aca.2020.09.051 33081941

[B81] Zhang T.-T. Xu J. Wang Y.-M. Xue C.-H. (2019). Health benefits of dietary marine dha/epa-enriched glycerophospholipids. Prog. lipid Res. 75, 100997. 10.1016/j.plipres.2019.100997 31442526

[B82] Zhang Y. Wu G. Zhang Y. Wang X. Jin Q. Zhang H. (2020). Advances in exogenous docosahexaenoic acid‐containing phospholipids: sources, positional isomerism, biological activities, and advantages. Compr. Rev. Food Sci. Food Saf. 19 (4), 1420–1448. 10.1111/1541-4337.12543 33337094

[B83] Zhou G.-Q. Wang X. Gao P. Qin T.-Z. Guo L. Zhang Z.-W. (2024). Intestinal microbiota via nlrp3 inflammasome dependent neuronal pyroptosis mediates anxiety-like behaviour in mice exposed to 3.5 ghz radiofrequency radiation. Sci. Total Environ. 927, 172391. 10.1016/j.scitotenv.2024.172391 38608899

[B84] Zhou L. Xiong J.-Y. Chai Y.-Q. Huang L. Tang Z.-Y. Zhang X.-F. (2022). Possible antidepressant mechanisms of omega-3 polyunsaturated fatty acids acting on the central nervous system. Front. Psychiatry 13, 933704. 10.3389/fpsyt.2022.933704 36117650 PMC9473681

[B85] Zhu W. Zhang Z. He S. Wong G. Banadyga L. Qiu X. (2018). Successful treatment of marburg virus with orally administrated t-705 (favipiravir) in a mouse model. Antivir. Res. 151, 39–49. 10.1016/j.antiviral.2018.01.011 29369776 PMC5844847

[B86] Zulu S. S. Abboussi O. Simola N. Mabandla M. V. Daniels W. M. U. (2021). Effects of combination antiretroviral drugs (cart) on hippocampal neuroplasticity in female mice. J. Neurovirol 27 (2), 325–333. 10.1007/s13365-021-00967-z 33710598

