# Supplementary material for: Dysregulated glycerophospholipid metabolism in amygdala may mediate favipiravir-induced anxiety-like behaviors in mice
Source: Front Pharmacol. 2025 Mar 4;16:1491150. doi: 10.3389/fphar.2025.1491150 (PMC11913839; doi:10.3389/fphar.2025.1491150)
Supplement: Supplementary file 1 [file DataSheet1.docx]

## Supplemental Information

## Supporting information includes:

## 3 Supplementary Figures

## 1 Supplementary Table

##
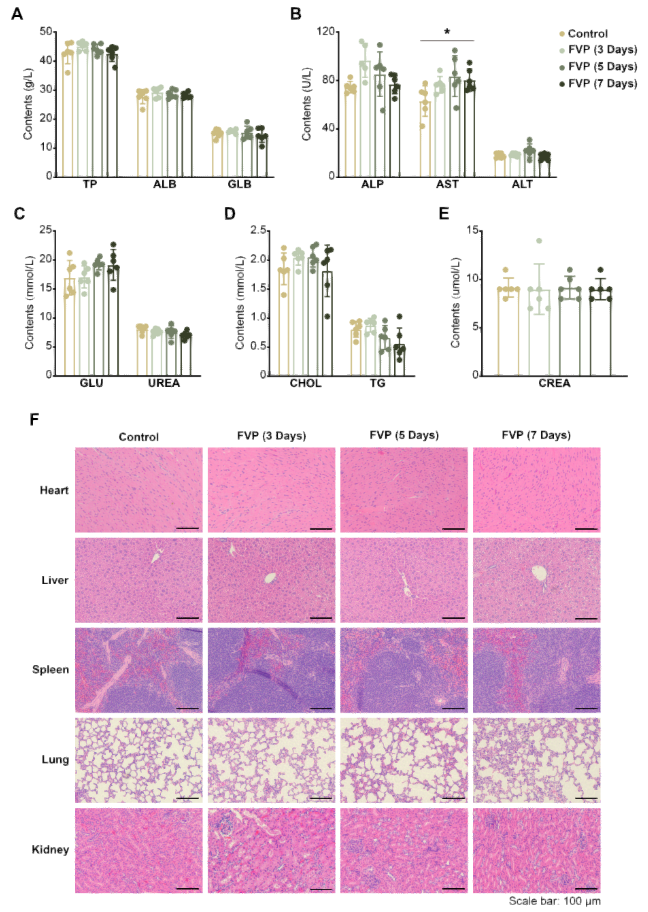


## Supplementary Fig. 1 (A-E) Changes of blood biochemical indexes in mice (AST: *p*=0.0362). Data from six mice (n=6). All data were presented as the mean ±SD; *, *p*<0.05; Dunnett's one-way ANOVA test. (F) Representative histological image of hearts, livers, spleens, lungs, and kidneys (HE staining) in mice. The image includes an enlargement bar (100 μm).


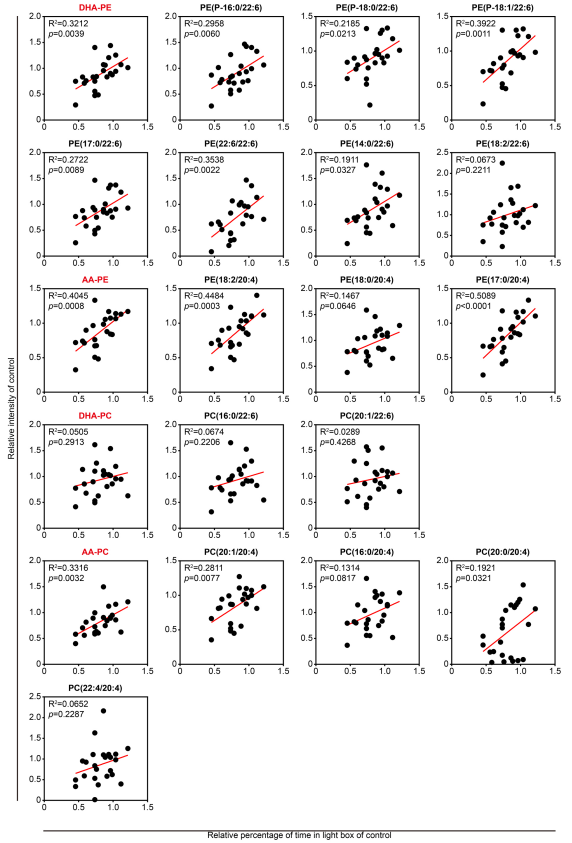


## Supplementary Fig. 2 In the dark/light box test, Pearson's test was used to analyze the correlation between DHA/AA-PE/PC abundance and percentage of time in light box. All Pearson correlation coefficient are shown in the figures.


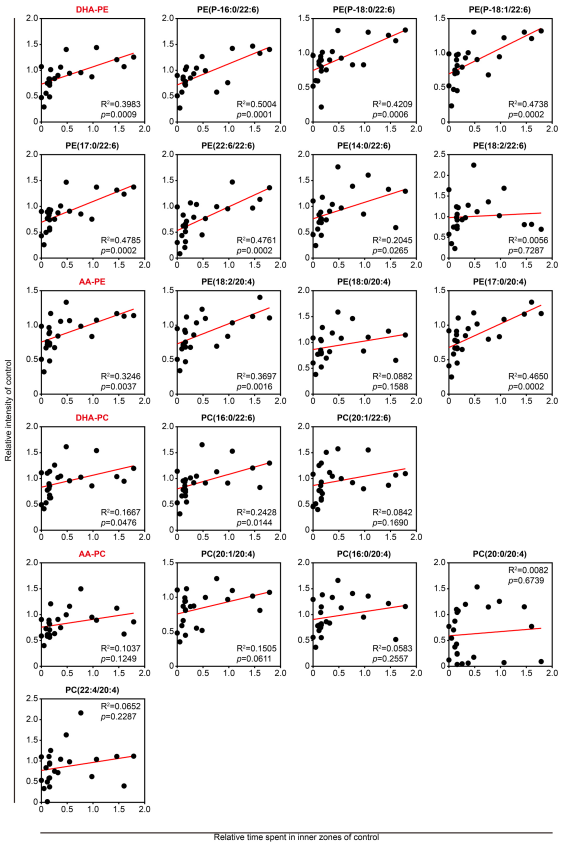


## Supplementary Fig. 3 In the open field test, Pearson's test was used to analyze the correlation between DHA/AA-PE/PC abundance and time spent in inner zones. All Pearson correlation coefficient are shown in the figure.


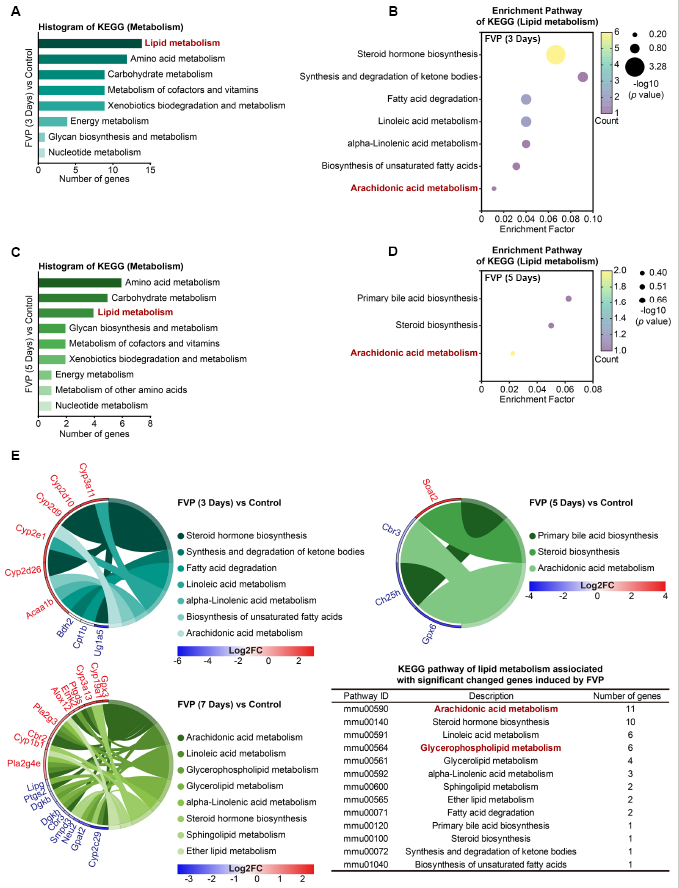


## Supplementary Fig. 4 (A, C) The KEGG pathway functional annotation analysis of metabolism related DEGs in the amygdala of mice after continuous treatment with favipiravir for 3 (A) and 5 days (C). (B, D) The KEGG pathway functional enrichment analysis of lipid metabolism-related DEGs in the amygdala of mice after continuous treatment with favipiravir for 3 (B) and 5 days (D). (E) The chord diagram of top significantly enriched KEGG pathways related to lipid metabolism. The right semicircle represents the name of the KEGG pathway, and the left semicircle is the name of the genes contained in the corresponding KEGG pathway. The order of gene display is arranged in descending order of log2FC. The larger log2FC, the larger the expression difference fold of the upregulated genes (red). The smaller the log2FC, the larger the expression difference fold of the downregulated genes (blue).


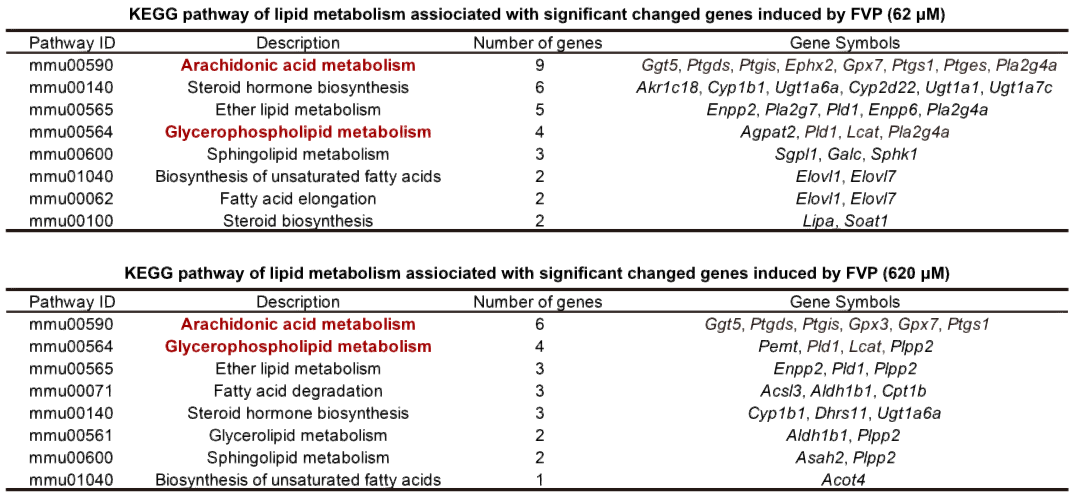


## Supplementary Fig. 5 The KEGG pathway of lipid metabolism related to the DEGs induced by fapiravir in the primary neurons.


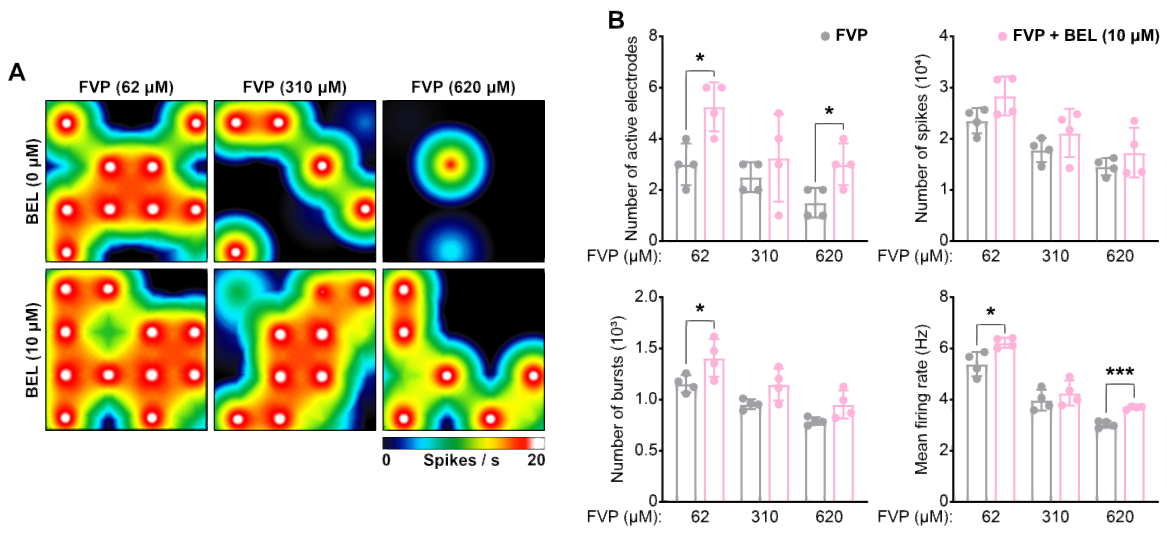


## Supplementary Fig. 6 (A) The heatmap of the representative real-time spike firing rate during MEA recording. The firing rate was color-coded, and the color transitions from white to black. (B) Quantification of the number of active electrodes, spikes, bursts and mean firing rate during 300 seconds of recording time. At least 4 wells (n=4) were chosen for analysis [Number of Active Electrodes: FVP (62 μM), *p*=0.0117, FVP (620 μM), *p*=0.0240; Number of Bursts: FVP (62 μM), *p*=0.0469; Mean Firing Rate: FVP (62 μM), *p*=0.0173, FVP (620 μM), *p*=0.0001]. All quantification data are presented as the mean±SD; *, *p*<0.05; **, *p*<0.01; ***, *p*<0.001; two-tailed *t* test.

**Supplementary Table 1. The exact *F* value, *t* value and *p* value for each figure.**

| **Fig.#** | | **Statistical method** | **n** | ***F* or *t* value** | ***p* value** |
| --- | --- | --- | --- | --- | --- |
| **1B** | One-way ANOVA | | 8 | *F*_treatment (3, 27)_=4.943 | *p*=0.0073 |
| **1E** | One-way ANOVA | | 8 | **Time spent in inner zones:**  *F*_treatment (3, 28)_=3.819  **Total disdance:**  *F*_treatment (3, 28)_=0.356 | **Time spent in inner zones:**  *p*=0.0206  **Total disdance:**  *p*=0.7850 |
| **1H** | One-way ANOVA | | 8 | **Immobile duration:**  *F*_treatment (3, 28)_=0.140  **Swim velocity:**  *F*_treatment (3, 28)_=0.400 | **Immobile duration:**  *p*=0.9355  **Swim velocity:**  *p*=0.7544 |
| **2F** | One-way ANOVA | | **6** | **Degree of unsaturation<5:**  *F*_treatment (3, 20)_=1.800  **Degree of unsaturation=5:**  *F*_treatment (3, 20)_=4.853  **Degree of unsaturation=6:**  *F*_treatment (3, 20)_=4.611  **Degree of unsaturation=7:**  *F*_treatment (3, 20)_=5.950  **Degree of unsaturation=8:**  *F*_treatment (3, 20)_=4.105  **Degree of unsaturation>8:**  *F*_treatment (3, 20)_=3.787 | **Degree of unsaturation<5:**  *p*=0.1796  **Degree of unsaturation=5:**  *p*=0.0107  **Degree of unsaturation=6:**  *p*=0.0131  **Degree of unsaturation=7:**  *p*=0.0045  **Degree of unsaturation=8:**  *p*=0.0201  **Degree of unsaturation>8:**  *p*=0.0267 |
| **2G** | One-way ANOVA | | **6** | **Chain length<39:**  *F*_treatment (3, 20)_=2.030  **Chain length=39:**  *F*_treatment (3, 20)_=3.552  **Chain length=40:**  *F*_treatment (3, 20)_=3.778  **Chain length>40:**  *F*_treatment (3, 20)_=1.612 | **Chain length<39:**  *p*=0.1420  **Chain length<39:**  *p*=0.0329  **Chain length<39:**  *p*=0.0269  **Chain length<39:**  *p*=0.2182 |
| **3B** | One-way ANOVA | | **6** | **PE:**  *F*_treatment (3, 20)_=1.718  **PC:**  *F*_treatment (3, 20)_=2.913  **PS:**  *F*_treatment (3, 20)_=1.382  **PG:**  *F*_treatment (3, 20)_=2.293  **PI:**  *F*_treatment (3, 20)_=0.260 | **PE:**  *p*=0.1954  **PC:**  *p*=0.0596  **PS:**  *p*=0.2770  **PG:**  *p*=0.1090  **PI:**  *p*=0.8536 |
|  | Two-tailed *t*-test | |  | **PE:**  3 D *vs* Control, *t*_(10)_=0.202  5 D *vs* Control, *t*_(10)_=0.871  7 D *vs* Control, *t*_(9)_=3.030  **PC:**  3 D *vs* Control, *t*_(10)_=0.287  5 D *vs* Control, *t*_(10)_=1.970  7 D *vs* Control, *t*_(10)_=2.526  **PS:**  3 D *vs* Control, *t*_(10)_=0.140  5 D *vs* Control, *t*_(10)_=0.225  7 D *vs* Control, *t*_(10)_=1.455  **PG:**  3 D *vs* Control, *t*_(10)_=0.487  5 D *vs* Control, *t*_(10)_=0.617  7 D *vs* Control, *t*_(10)_=1.927  **PI:**  3 D *vs* Control, *t*_(10)_=0.260  5 D *vs* Control, *t*_(10)_=0.162  7 D *vs* Control, *t*_(10)_=0.689 | **PE:**  3 D *vs* Control, *p*=0.8444  5 D *vs* Control, *p*=0.4043  7 D *vs* Control, *p*=0.0143  **PC:**  3 D *vs* Control, *p*=0.7800  5 D *vs* Control, *p*=0.0772  7 D *vs* Control, *p*=0.0301  **PS:**  3 D *vs* Control, *p*=0.8920  5 D *vs* Control, *p*=0.8268  7 D *vs* Control, *p*=0.1764  **PG:**  3 D *vs* Control, *p*=0.6365  5 D *vs* Control, *p*=0.5511  7 D *vs* Control, *p*=0.0828  **PI:**  3 D *vs* Control, *p*=0.8002  5 D *vs* Control, *p*=0.8748  7 D *vs* Control, *p*=0.5067 |
| **3C** | One-way ANOVA | | **6** | **PE(16:0/20:3):**  *F*_treatment (3, 20)_=3.916  **PE(P-16:0/22:6):**  *F*_treatment (3, 20)_=3.414  **PE(18:0/20:4):**  *F*_treatment (3, 20)_=4.156  **PE(P-18:0/22:6):**  *F*_treatment (3, 20)_=4.547  **PE(20:0/18:1):**  *F*_treatment (3, 20)_=3.217  **PE(P-18:1/22:6):**  *F*_treatment (3, 20)_=5.538  **PE(38:4):**  *F*_treatment (3, 20)_=4.659  **PE(18:0/20:4):**  *F*_treatment (3, 20)_=2.496 | **PE(16:0/20:3):**  *p*=0.0238  **PE(P-16:0/22:6):**  *p*=0.0373  **PE(18:0/20:4):**  *p*=0.0193  **PE(P-18:0/22:6):**  *p*=0.0138  **PE(20:0/18:1):**  *p*=0.0448  **PE(P-18:1/22:6):**  *p*=0.0062  **PE(38:4):**  *p*=0.0126  **PE(18:0/20:4):**  *p*=0.0892 |
| **3D** | One-way ANOVA | | **6** | **PE(17:0):**  *F*_treatment (3, 20)_=2.850  **PE(17:0/20:4):**  *F*_treatment (3, 20)_=5.384  **PE(17:0/22:6):**  *F*_treatment (3, 20)_=3.703  **PE(P-18:1/23:1):**  *F*_treatment (3, 20)_=2.634  **PE(22:6/22:6):**  *F*_treatment (3, 20)_=9.420  **PE(18:2/22:6):**  *F*_treatment (3, 20)_=2.106  **PE(18:1/16:0):**  *F*_treatment (3, 20)_=1.358  **PE(14:0/22:6)**  *F*_treatment (3, 20)_=3.323 | **PE(17:0):**  *p*=0.0633  **PE(17:0/20:4):**  *p*=0.0070  **PE(17:0/22:6):**  *p*=0.0287  **PE(P-18:1/23:1):**  *p*=0.0779  **PE(22:6/22:6):**  *p*=0.0004  **PE(18:2/22:6):**  *p*=0.1315  **PE(18:1/16:0):**  *p*=0.2842  **PE(14:0/22:6):**  *p*=0.0406 |
| **3E** | One-way ANOVA | | **6** | **PC(40:7):**  *F*_treatment (3, 20)_=3.261  **PC(38:5):**  *F*_treatment (3, 20)_=4.474  **PC(16:0/18:1):**  *F*_treatment (3, 20)_=3.794  **PC(34:0):**  *F*_treatment (3, 20)_=3.365  **PC(32:0):**  *F*_treatment (3, 20)_=3.086  **PC(38:6):**  *F*_treatment (3, 20)_=3.578  **PC(36:4):**  *F*_treatment (3, 20)_=2.463  **PC(16:0/22:6):**  *F*_treatment (3, 20)_=2.918 | **PC(40:7):**  *p*=0.0429  **PC(38:5):**  *p*=0.0147  **PC(16:0/18:1):**  *p*=0.0265  **PC(34:0):**  *p*=0.0390  **PC(32:0):**  *p*=0.0506  **PC(38:6):**  *p*=0.0321  **PC(36:4):**  *p*=0.0922  **PC(16:0/22:6):**  *p*=0.0593 |
| **3F** | One-way ANOVA | | **6** | **PC(19:1):**  *F*_treatment (3, 20)_=4.920  **PC(20:1/20:4):**  *F*_treatment (3, 20)_=6.400  **PC(20:1/22:6):**  *F*_treatment (3, 20)_=4.626  **PC(18:0/22:5):**  *F*_treatment (3, 20)_=4.693  **PC(16:0/20:4):**  *F*_treatment (3, 20)_=2.943  **PC(20:0/20:4):**  *F*_treatment (3, 20)_=5.081  **PC(22:4/20:4):**  *F*_treatment (3, 20)_=1.172  **PC(16:1/16:1):**  *F*_treatment (3, 20)_=8.174 | **PC(19:1):**  *p*=0.0101  **PC(20:1/20:4):**  *p*=0.0032  **PC(20:1/22:6):**  *p*=0.0129  **PC(18:0/22:5):**  *p*=0.0122  **PC(16:0/20:4):**  *p*=0.0579  **PC(20:0/20:4):**  *p*=0.0089  **PC(22:4/20:4):**  *p*=0.3452  **PC(16:1/16:1):**  *p*=0.0010 |
| **4G** | One-way ANOVA | | **3** | ***Gpat2*:**  *F*_treatment (3, 6)_=5.362  ***Pla2g4e*:**  *F*_treatment (3, 5)_=6.680  ***Ptgds*:**  *F*_treatment (3, 8)_=19.920  ***Cbr2*:**  *F*_treatment (3, 8)_=6.144 | ***Gpat2*:**  *p*=0.0391  ***Pla2g4e*:**  *p*=0.0336  ***Ptgds*:**  *p*=0.0005  ***Cbr2*:**  *p*=0.0180 |
| **6C** | One-way ANOVA | | **20** | *F*_treatment (2, 57)_=29.750 | *p*<0.0001 |
| **6D** | One-way ANOVA | | **20** | *F*_treatment (2, 57)_=20.930 | *p*<0.0001 |
| **6F** | One-way ANOVA | | **20** | *F*_treatment (2, 57)_=41.180 | *p*<0.0001 |
| **7C** | One-way ANOVA | | **3** | **Number of Active Electrodes:**  *F*_treatment (3, 8)_=5.241  **Number of Spikes:**  *F*_treatment (3, 8)_=14.520  **Number of Bursts:**  *F*_treatment (3, 8)_=28.430  **Mean Firing Rate:**  *F*_treatment (3, 8)_=14.520  **Network Burst Frequency:**  *F*_treatment (3, 8)_=43.220  **Network Burst Percentage:**  *F*_treatment (3, 8)_=11.200 | **Number of Active Electrodes:**  *p*=0.0272  **Number of Spikes:**  *p*=0.0013  **Number of Bursts:**  *p*=0.0001  **Mean Firing Rate:**  *p*=0.0013  **Network Burst Frequency:**  *p*<0.0001  **Network Burst Percentage:**  *p*=0.0031 |
| **7D** | One-way ANOVA | | **4** | **Number of Active Electrodes:**  *F*_treatment (3, 12)_=5.226  **Number of Spikes:**  *F*_treatment (3, 12)_=5.741  **Number of Bursts:**  *F*_treatment (3, 12)_=3.277  **Mean Firing Rate:**  *F*_treatment (3, 12)_=20.310  **Network Burst Frequency:**  *F*_treatment (3, 12)_=14.580  **Network Burst Percentage:**  *F*_treatment (3, 12)_=2.902 | **Number of Active Electrodes:**  *p*=0.0154  **Number of Spikes:**  *p*=0.0113  **Number of Bursts:**  *p*=0.0587  **Mean Firing Rate:**  *p*<0.0001  **Network Burst Frequency:**  *p*=0.0003  **Network Burst Percentage:**  *p*=0.0787 |
| **S1A** | One-way ANOVA | | **6** | **TP:**  *F*_treatment (3, 20)_=1.308  **ALB:**  *F*_treatment (3, 20)_=0.932  **GLB:**  *F*_treatment (3, 20)_=0.672 | **TP:**  *p*=0.2996  **ALB:**  *p*=0.4435  **GLB:**  *p*=0.5790 |

| **S1B** | One-way ANOVA | **6** | **ALP:**  *F*_treatment (3, 19)_=3.523  **AST:**  *F*_treatment (3, 19)_=3.446  **ALT:**  *F*_treatment (3, 20)_=2.734 | **ALP:**  *p*=0.0349  **AST:**  *p*=0.0362  **ALT:**  *p*=0.0707 |
| --- | --- | --- | --- | --- |
| **S1C** | One-way ANOVA | **6** | **GLU:**  *F*_treatment (3, 20)_=1.902  **UREA:**  *F*_treatment (3, 20)_=2.079 | **GLU:**  *p*=0.1618  **UREA:**  *p*=0.1351 |
| **S1D** | One-way ANOVA | **6** | **CHOL:**  *F*_treatment (3, 20)_=1.214  **TG:**  *F*_treatment (3, 20)_=3.322 | **CHOL:**  *p*=0.3302  **TG:**  *p*=0.0406 |
| **S1E** | One-way ANOVA | **6** | *F*_treatment (3, 20)_=0.022 | *p*=0.9956 |
| **S6B** | One-way ANOVA | **4** | **Number of Active Electrodes:**  FVP (62 μM):  BEL *vs* non-BEL, *t*_(6)_=3.576  FVP (310 μM):  BEL *vs* non-BEL, *t*_(6)_=0.832  FVP (620 μM):  BEL *vs* non-BEL, *t*_(6)_=3.000  **Number of Spikes:**  FVP (62 μM):  BEL *vs* non-BEL, *t*_(6)_=2.132  FVP (310 μM):  BEL *vs* non-BEL, *t*_(6)_=1.257  FVP (620 μM):  BEL *vs* non-BEL, *t*_(6)_=1.069  **Number of Bursts:**  FVP (62 μM):  BEL *vs* non-BEL, *t*_(6)_=2.495  FVP (310 μM):  BEL *vs* non-BEL, *t*_(6)_=2.365  FVP (620 μM):  BEL *vs* non-BEL, *t*_(6)_=2.307  **Mean Firing Rate:**  FVP (62 μM):  BEL *vs* non-BEL, *t*_(6)_=3.259  FVP (310 μM):  BEL *vs* non-BEL, *t*_(6)_=0.8704  FVP (620 μM):  BEL *vs* non-BEL, *t*_(6)_=8.914 | **Number of Active Electrodes:**  FVP (62 μM):  BEL *vs* non-BEL, *p*=0.0117  FVP (310 μM):  BEL *vs* non-BEL, *p*=0.4372  FVP (620 μM):  BEL *vs* non-BEL, *p*=0.0240  **Number of Spikes:**  FVP (62 μM):  BEL *vs* non-BEL, *p*=0.0770  FVP (310 μM):  BEL *vs* non-BEL, *p*=0.2553  FVP (620 μM):  BEL *vs* non-BEL, *p*=0.3262  **Number of Bursts:**  FVP (62 μM):  BEL *vs* non-BEL, *p*=0.0469  FVP (310 μM):  BEL *vs* non-BEL, *p*=0.0559  FVP (620 μM):  BEL *vs* non-BEL, *p*=0.0605  **Mean Firing Rate:**  FVP (62 μM):  BEL *vs* non-BEL, *p*=0.0173  FVP (310 μM):  BEL *vs* non-BEL, *p*=0.4176  FVP (620 μM):  BEL *vs* non-BEL, *p*=0.0001 |
